# The Merkel Cell Polyomavirus Minor Capsid Protein

**DOI:** 10.1371/journal.ppat.1003558

**Published:** 2013-08-22

**Authors:** Rachel M. Schowalter, Christopher B. Buck

**Affiliations:** Tumor Virus Molecular Biology Section, Laboratory of Cellular Oncology, Center for Cancer Research, National Cancer Institute, National Institutes of Health, Bethesda, Maryland, United States of America; University of Michigan, United States of America

## Abstract

The surface of polyomavirus virions is composed of pentameric knobs of the major capsid protein, VP1. In previously studied polyomavirus species, such as SV40, two interior capsid proteins, VP2 and VP3, emerge from the virion to play important roles during the infectious entry process. Translation of the VP3 protein initiates at a highly conserved Met-Ala-Leu motif within the VP2 open reading frame. Phylogenetic analyses indicate that Merkel cell polyomavirus (MCV or MCPyV) is a member of a divergent clade of polyomaviruses that lack the conserved VP3 N-terminal motif. Consistent with this observation, we show that VP3 is not detectable in MCV-infected cells, VP3 is not found in native MCV virions, and mutation of possible alternative VP3-initiating methionine codons did not significantly affect MCV infectivity in culture. In contrast, VP2 knockout resulted in a >100-fold decrease in native MCV infectivity, despite normal virion assembly, viral DNA packaging, and cell attachment. Although pseudovirus-based experiments confirmed that VP2 plays an essential role for infection of some cell lines, other cell lines were readily transduced by pseudovirions lacking VP2. In cell lines where VP2 was needed for efficient infectious entry, the presence of a conserved myristoyl modification on the N-terminus of VP2 was important for its function. The results show that a single minor capsid protein, VP2, facilitates a post-attachment stage of MCV infectious entry into some, but not all, cell types.

## Author Summary

Merkel cell polyomavirus (MCV or MCPyV) is a recently discovered member of the viral family *Polyomaviridae*. The virus plays a causal role in Merkel cell carcinoma, a highly lethal form of skin cancer. MCV encodes a major capsid protein, VP1, which forms the non-enveloped surface of the virion. Other polyomavirus species encode two minor capsid proteins, VP2 and VP3, which associate with the inner surface of the capsid and facilitate infectious entry. In this report we show that MCV does not have a VP3 minor capsid protein. Sequence analyses suggest that more than a quarter of known polyomavirus species share MCV's lack of a VP3 protein. In contrast to VP3, VP2-knockout MCV mutants displayed dramatically reduced infectivity. Consistent with native virion findings, MCV pseudovirions lacking VP2 or carrying mutations in the VP2 myristoylation motif displayed reduced infectivity on several cell lines. Puzzlingly, MCV pseudoviruses lacking VP2 successfully transduced other cell lines with high efficiency. Taken together, the data show that the lone MCV minor capsid protein, VP2, plays an important role during infectious entry into some cell types, but is dispensable for entry into other cell types.

## Introduction

The icosahedral polyomavirus capsid is constructed from 72 pentameric capsomers of the major capsid protein, VP1 [1]. VP1 mediates attachment of the virus to cell surface glycan receptors [2], [3], initiating infectious entry and delivery of the encapsidated circular ∼5 kb dsDNA viral genome to host cells. The VP1 protein of previously studied polyomaviruses, such as simian virus 40 (SV40) and murine polyomavirus (MPyV), associates with two minor capsid proteins, called VP2 and VP3, that are thought to emerge from the capsid interior to play important roles during the infectious entry process [4]–[7]. VP2 and VP3 are translated from a single, un-spliced open reading frame (ORF) [8]. The initiating AUG codon of VP3 is in-frame with and downstream of the initiating VP2 AUG. Thus, VP3 is an N-truncated isoform of VP2. In most polyomaviruses, the unique N-terminus of VP2 carries a consensus sequence for N-terminal myristoylation (MGXXXS/T). The VP2 proteins of SV40 and MPyV have been shown to be myristoylated, and mutations that prevent myristoylation reduce the infectivity of MPyV [9]–[11].

In 2008, a new human polyomavirus was discovered in a rare and aggressive form of skin cancer, known as Merkel cell carcinoma (MCC) [12]. The virus was named Merkel cell polyomavirus (MCV or MCPyV). A large number of studies indicate that accidental integration of MCV DNA into the host genome is a causal factor in most cases of MCC [13], [14]. Although MCC is uncommon, chronic MCV infection of human skin appears to be very common, and most infected people will experience no known symptoms as a result of their infection with MCV [15], [16]. Since the discovery of MCV, renewed interest in polyomaviruses and advances in deep sequencing technologies have led to the discovery of many additional human and animal polyomaviruses [17]–[35]. The newly expanded view of this viral family permits greater resolution of significant sequence differences between members. For instance, alignment of VP2 sequences reveals that a subset of polyomaviruses, including MCV, lack a conserved peptide motif that comprises the N-terminus of known VP3 proteins. The VP1 and VP2 proteins of this subset of polyomaviruses have in common other distinctive sequence features, which will be discussed in detail below. These sequence differences led us to question whether the expression and function of the MCV minor capsid proteins might differ from more extensively studied members of the polyomavirus family.

Several publications have addressed the role of VP2 and VP3 in the life cycle of different polyomaviruses. However, it is difficult to draw universal conclusions about the precise function of the minor capsid proteins from these reports. Results consistent with a defect in virion assembly and viral genome packaging were reported when one or both minor capsid proteins of JC polyomavirus (JCV or JCPyV) were deleted [36]. In contrast, SV40 VP1-only virions packaged and protected viral DNA as efficiently as VP1/2/3 virions [4], [5]. SV40 attachment to cell surfaces was determined to be normal in the absence of both minor capsid proteins in one report [4], but binding of VP2-deleted SV40 virions appeared reduced in another report [5]. In nearly every case, infection or plaque formation by the virus is reduced or abrogated when either of the minor capsid proteins is deleted [4]–[6], [11], [36], [37], but the mechanism by which the minor capsid proteins facilitate infection remains controversial. The work of Daniels et al. [5] suggests that the role of both SV40 minor capsid proteins is to promote escape of the virus from the endoplasmic reticulum (ER). Inoue et al. [6] agreed that VP3 was needed for ER escape, but concluded that the principal role of VP2 was to direct trafficking of the virus to the ER. Conversely, Nakanishi et al. [4] provided evidence that the role of the SV40 minor capsid proteins lay in nuclear import of the viral genome during the infectious entry process and found that the virus could escape from the ER into the cytoplasm without the benefit of minor capsid proteins. Reports concerning the role and importance of VP2 myristoylation also lack uniformity [9], [11], [36]–[38].

We have examined, for the first time, the expression and function of the MCV VP2 protein as well as the previously proposed VP3 protein of MCV using both pseudoviruses and native MCV virions propagated in cell culture. VP3 protein was not detected in MCV-infected cells or in purified MCV virions. Our phylogenetic analyses suggest that this feature of MCV is typical of an emerging clade of polyomaviruses.

## Results

### The Absence of an MCV VP3 Protein

The late 19S SV40 mRNA was previously shown to code for multiple proteins through a mechanism of “leaky” ribosomal scanning [8], [39]. The protein products of the SV40 19S mRNA can be predicted based on what is known of translation initiation at Kozak consensus sequences. The optimal Kozak consensus sequence is minimally defined as GCC **R**CC AUG **G** (where the most important positions are in bold) [40]. The relatively weak Kozak sequence surrounding the SV40 VP2 translation initiation site (AGG **U**CC AUG **G**) is thought to result in it being bypassed by scanning ribosomes with a frequency of about 70%, while the stronger Kozak context of VP3 (CCA **G**GA AUG **G**) results in more frequent translational initiation of this gene product [39]. In contrast to SV40, the MCV VP2 Kozak sequence is strong (UUC **A**GG AUG **G**) and the Kozak context surrounding the proposed MCV VP3 initiation codon (Met_46_) [12] is very weak (AGU **U**UA AUG **A**). The Kozak sequence surrounding the only other methionine codon in VP2 (Met_129_) is weak (GCA **C**UU AUG **G**), and ribosome access to Met_129_ is also obstructed by four out-of-frame upstream AUG codons (in addition to Met_1_ and Met_46_). Since conventionally scanning ribosomes would initially encounter the strong Kozak sequence surrounding the VP2 initiation site before they could reach either of the weaker initiation codons that might conceivably initiate a VP3-like protein, VP2 seems likely to be the primary product of the MCV late mRNA. An additional consideration in our sequence-based analyses is that documented VP3 proteins carry a consensus N-terminal motif defined as MALXXΦ, where Φ represents an aromatic residue. MCV VP2 does not encode any sequence with homology to this conserved VP3 N-terminal motif (Figure S1).

**Figure 1:**
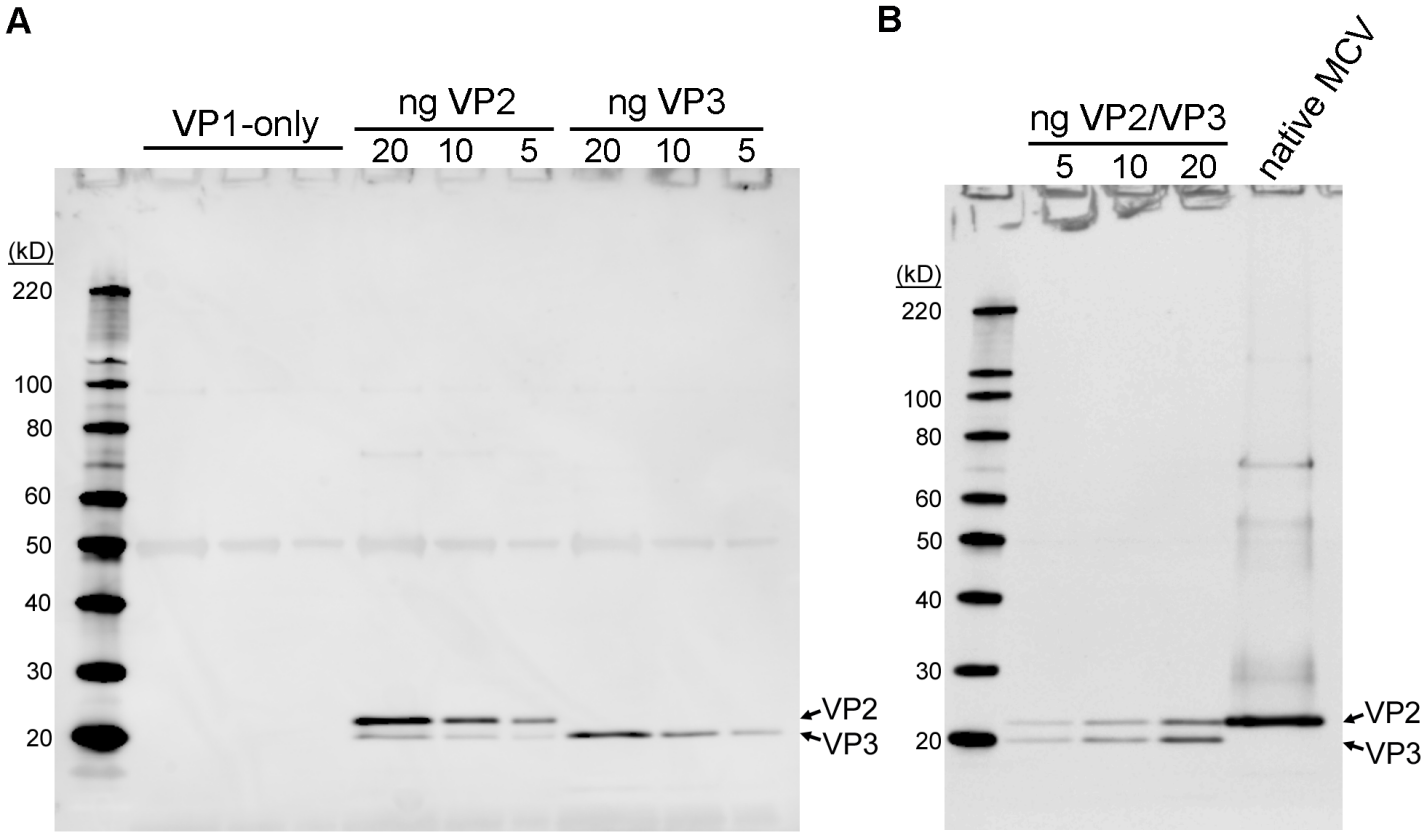
Native MCV virions do not contain detectable VP3 proteins. (A) Pseudovirions containing known amounts (indicated; units of ng/lane) of VP2 or putative VP3 protein or a similar amount of VP1-only pseudovirions were western blotted using polyclonal anti-VP2 serum. (B) Native MCV virions immunoprecipitated with anti-VP1 monoclonal antibodies were western blotted with anti-VP2 rabbit serum alongside an MCV VP1/2/3 pseudovirion standard (amount of VP3 is indicated; units of ng/lane).

We have previously published methods for production of native MCV virions from 293TT cells transfected with MCV genomic DNA [41], [42]. Purified MCV virions can be propagated in a cell line named 293-4T, which stably expresses the MCV small t (sT) and Large T (LT) antigens. The 293TT system can also be used for intracellular production of recombinant MCV-based reporter vectors (pseudovirions) [15]. Using these tools, we sought to determine the minor capsid protein make-up of MCV virions. When purified MCV virions produced by transfection of 293TT cells were initially examined by SDS-PAGE and western blot with a rabbit polyclonal anti-VP2 antiserum, VP2 could readily be detected but no proteins migrating faster than VP2 were apparent (data not shown). To increase the possibility of detecting a VP3 protein that was simply not abundant, larger amounts of 293-4T-propagated native MCV virions were concentrated by immunoprecipitation using an anti-VP1 monoclonal antibody. The concentrated native virions were compared to recombinant pseudovirion standards produced with or without VP2 and VP3 (Figure 1A). Western blotting of concentrated native virions showed a strong VP2 band and no visible VP3 bands (Figure 1B). Thus, MCV virions produced using the native viral regulatory sequences do not contain detectable amounts of VP3 capsid protein.

Analysis of recombinant pseudovirions shows that recombinantly expressed VP3 protein is stably incorporated into VP1 capsids (Figure 1A). The absence of VP3 proteins in native MCV virions thus presumably reflects a lack of VP3 protein expression in MCV-infected 293-4T cells. When whole lysates of 293-4T cells transfected with MCV genomic DNA were analyzed by western blot with VP2 antiserum, VP3 proteins were again not visible, confirming that MCV does not express detectable amounts of VP3 protein (Figure S2).

**Figure 2:**
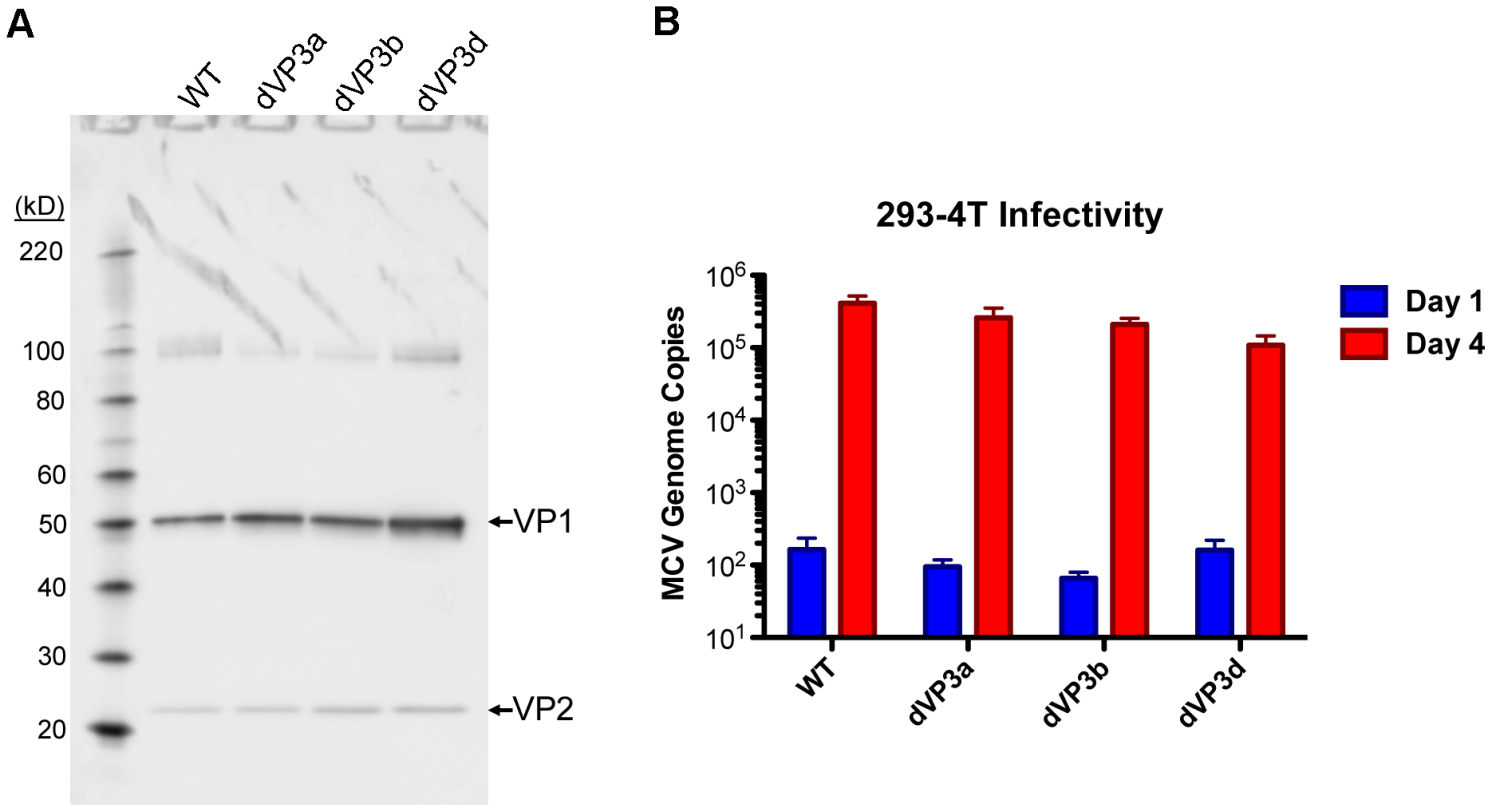
Mutation of VP2 internal methionine residues has little effect on MCV infectivity. (A) Purified native MCV carrying mutations in the first internal methionine (Met_46_) of VP2 (dVP3a), the second internal methionine (Met_129_) of VP2 (dVP3b) or a double Met mutant (dVP3d) were analyzed alongside WT MCV by SDS-PAGE and western blot with a mixture of anti-VP1 and anti-VP2 rabbit sera. These native virus stocks were not produced simultaneously and differed slightly in concentration. Four µl of WT and dVP3d and 2 µl of dVP3a and dVP3b were run on the gel. (B) Equal amounts of WT and dVP3 mutant viruses, normalized by MCV genomic copies, were inoculated onto 293-4T cells. Samples taken one day or four days post-infection were analyzed for viral DNA concentration by qPCR to determine replication of virus-delivered genomic DNA as a measure of infectivity. The average of three experiments performed in duplicate is shown and error bars represent the standard error of the mean.

In an effort to genetically rule out the possibility that undetectably low levels of VP3 might play a role in MCV infection, both of the internal VP2-frame methionine codons (Met_46_ and Met_129_) were mutated in the context of the MCV genome. Based on homology to the VP2 proteins of close relatives of MCV, Met_46_ was changed to a valine (mutant dVP3a) and Met_129_ was changed to a leucine (mutant dVP3b). A third variant was created (dVP3d) in which both methionine residues were mutated. Purified virion preparations for each of the mutants were produced and their infectivity was compared to that of a preparation of wild-type (WT) MCV virions. The yield of each virus was similar, there was a similar ratio of VP2 to VP1, and the number of MCV genomes packaged was nearly equivalent in each of the viruses (Figure 2A and data not shown). When the WT or mutant viral genomes were transfected into 293-4T cells, there was no evidence that the mutations affected VP1 or VP2 expression or altered cell viability (Figure S2 and S3). The infectivity of the viruses was assessed in 293-4T cells, which were inoculated with equivalent amounts of each virus, standardized by genomic copies (10 viral genomes/cell). Viral genome replication was determined by quantitative PCR (qPCR). Individual mutation of the VP3a and VP3b methionine residues in native MCV virions resulted in less than a two-fold decrease in infectivity of the viruses (Figure 2B). The apparent infectivity of the double mutant was reduced by about four-fold, suggesting the effect of the mutations was additive. Unfortunately, it is not possible to determine if the modest reduction in infectivity is a consequence of mutating the VP2 protein (causing VP2 to function less effectively) or if the WT virus expresses an undetectable amount of VP3 that confers a minor improvement in infectivity. Nevertheless, these results show that both conceivable VP3 proteins are largely or entirely dispensable for native MCV infection.

MCV reporter pseudoviruses were used to confirm the native virion results. Production of pseudovirions is less costly and less time-consuming than native virion production, assays for pseudovirion transduction have much higher throughput, and pseudovirions can transduce a wide variety of cell lines that do not support native MCV replication. The pseudovirus system also allows ectopic expression of candidate VP3 proteins. We have previously reported that supplementing the pseudovirus production system with VP3 expression plasmids does not improve the infectivity of MCV reporter pseudovirions [15]. However, the VP2 expression construct used in the previous report was intentionally designed to have a poor Kozak sequence context surrounding VP2, allowing limited amounts of VP3 expression by leaky scanning (Figure 1A). In an initial set of pseudovirus-based experiments, we found that improving the Kozak context surrounding the recombinant VP2-initiating AUG codon reduced the amount of “leaky” VP3 expression to undetectable levels (data not shown). Despite the reduced VP3 expression, pseudoviruses made using the improved-Kozak VP2 expression construct transduced 293TT cells with efficiency similar to pseudoviruses made using the original leaky VP2 expression construct (data not shown).

**Figure 3:**
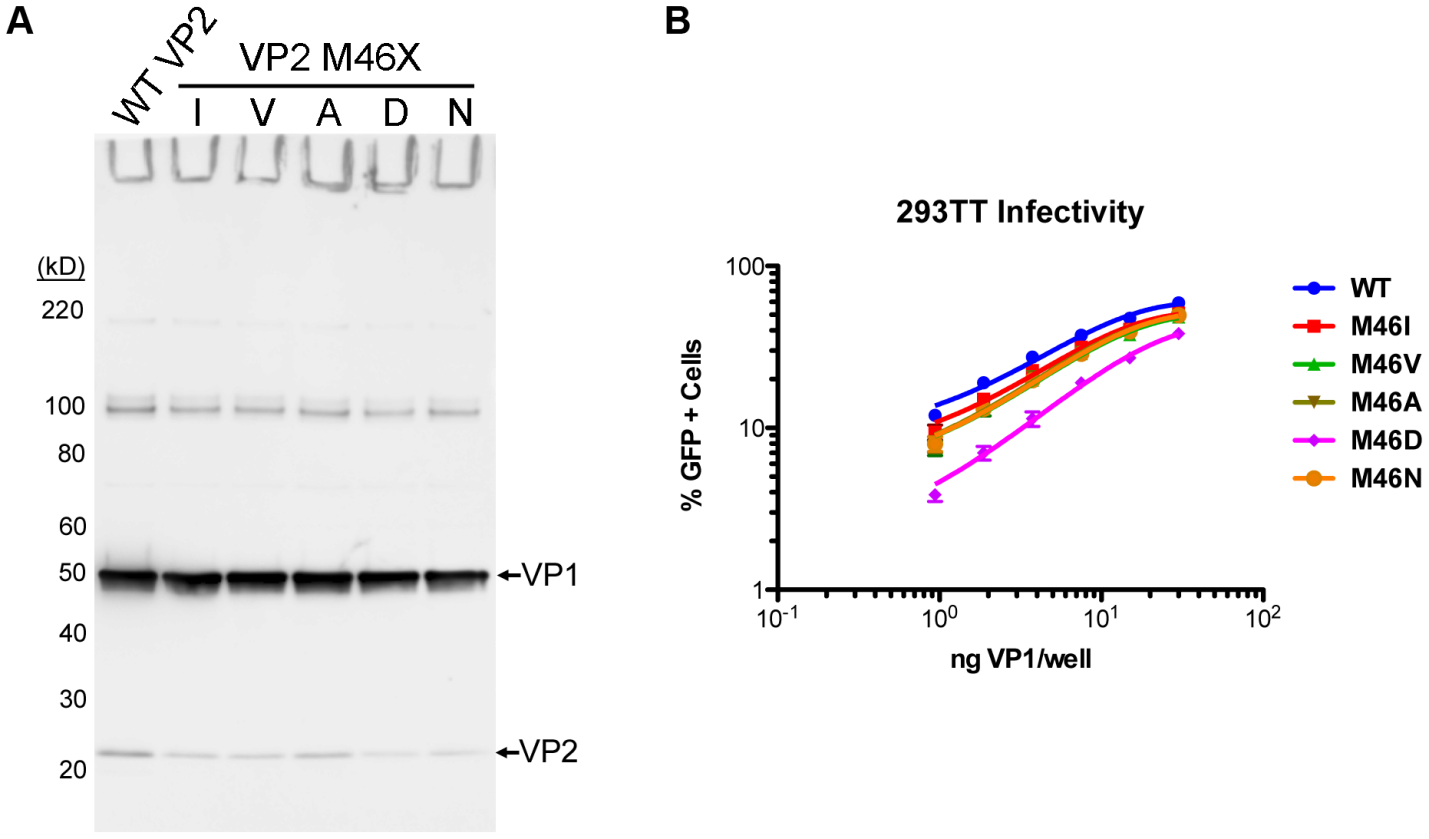
The effect of VP3 mutation on MCV pseudovirus transduction. (A) Clarified cell lysate containing MCV pseudovirions produced with WT or Met_46_ mutants of VP2 (M46I, M46V, M46A, M46D, or M46N) were analyzed by SDS-PAGE and western blot with a mixture of anti-VP1 and anti-VP2 rabbit sera. (B) The infectivity of the WT and M46X mutant pseudoviruses was examined in 293TT cells by flow cytometry. The average percentage of GFP-positive cells from three experiments is shown and error bars represent the standard error of the mean.

To eradicate VP3 expression, the proposed VP3-initiating Met_46_ was comprehensively mutated to various other amino acids, anticipating that some VP2 mutations might be better tolerated than others. A confounding variable in these experiments was that some mutant VP2 proteins appeared to be expressed at a lower level than WT VP2, while VP1 expression was similar (Figure 3A). The transduction efficiency of WT and mutant pseudovirions, normalized to VP1 concentration, was determined in 293TT cells (Figure 3B). The majority of Met_46_ mutants (Ile, Val, Ala, and Asn) exhibited a ∼20–30% reduction in infectivity, while transduction by the M46D mutant was reduced by ∼65%. This shows that some Met_46_ mutations have a modest impact on VP2 biology and confirms that eradication of VP3 expression has little or no effect on pseudovirion infectivity.

### The Role and Importance of the MCV VP2 Protein

**Figure 4:**
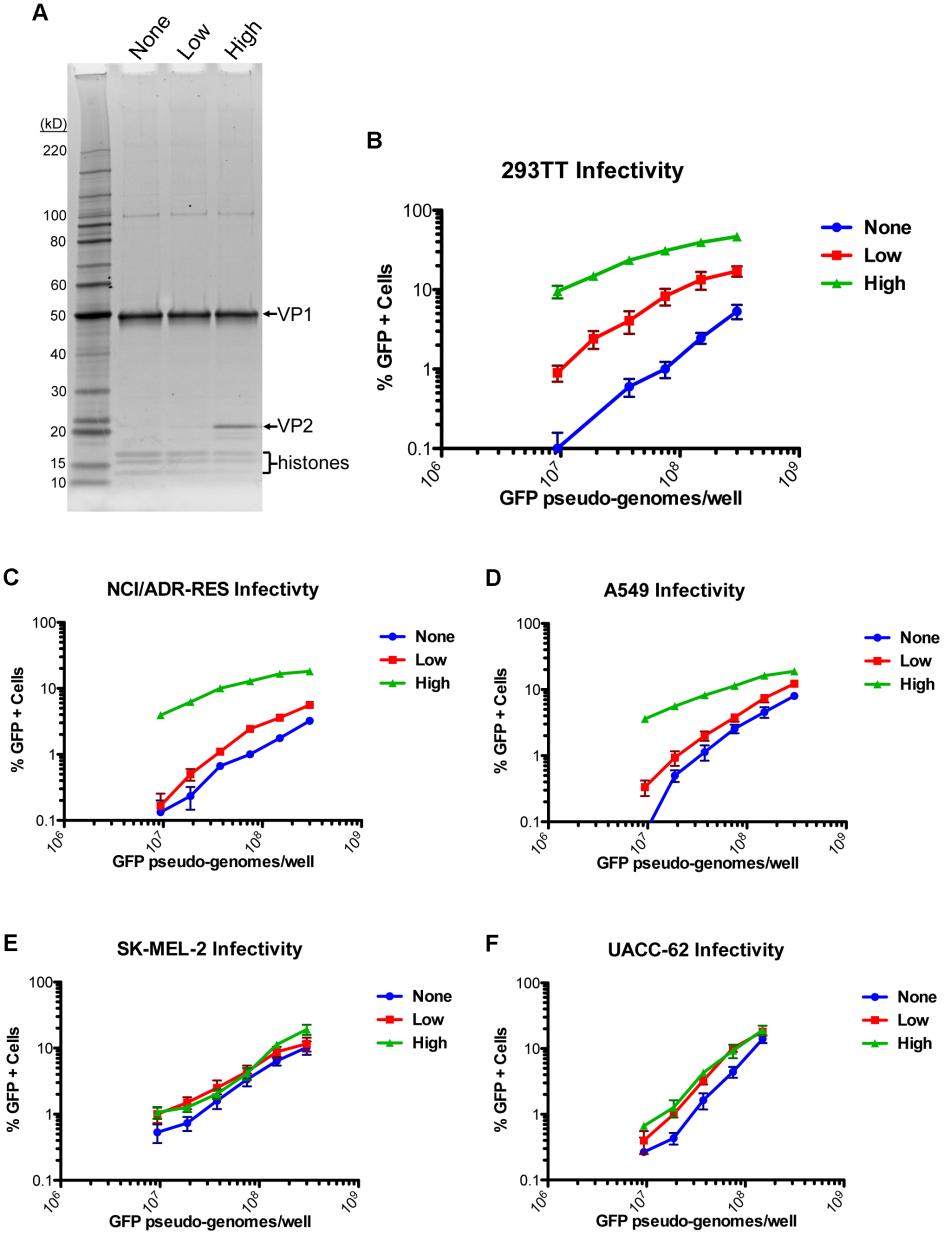
Differing effects of the MCV VP2 protein on pseudovirus transduction. (A) Purified MCV pseudovirions with no VP2 (None) a low level of VP2 (Low) or a high level of VP2 (High) were analyzed by SDS-PAGE and SYPRO Ruby protein stain. (B) The infectivity of MCV pseudoviruses with differing levels of VP2 was analyzed in 293TT cells by flow cytometry. The average percentage of GFP-positive cells from three experiments is shown and error bars represent the standard error of the mean. (C–F) Analysis of transduction as above, in other cell lines previously shown to be highly transducible with MCV VP1/VP2 pseudoviruses.

The data show that MCV is distinct from previously studied polyomaviruses in that it does not employ a VP3 minor capsid protein. In light of this discovery, the importance of the VP2 protein was next explored. MCV pseudovirions were produced by expression of VP1 in the absence of VP2 (“None”), with a low level of VP2 (“Low”), or with a high level of VP2 (“High”). The difference in VP2 incorporation in purified pseudovirus preparations can be observed in a protein stain of denatured pseudovirions in SDS-PAGE (Figure 4A). Quantitative PCR analysis of the encapsidated reporter plasmid associated with each pseudovirus preparation suggested that VP2 had no role in the encapsidation of the reporter plasmid (data not shown). When pseudovirion stocks were normalized by reporter gene copy number and inoculated onto 293TT cells, a VP2 dose-dependent effect on infectivity was observed (Figure 4B). Of note, transduction of cells by the VP1-only particles was also non-zero and VP1 dose-dependent.

To verify that VP2 plays an important role in efficient MCV entry in other cell types, several additional cell lines that had previously been shown to be highly transducible by MCV [42] were challenged with pseudovirions containing varying levels of VP2. Intriguingly, the effect of VP2 on MCV pseudovirus transduction efficiency differed dramatically from one cell type to the next (Figure 4C–F). Transduction of NCI/ADR-RES cells (an ovarian cancer line) was strongly enhanced by high levels of VP2, but low levels of VP2 improved transduction very little relative to VP1-only pseudoviruses. Transduction of A549 cells (a lung cancer line) was also improved by the presence of VP2, but the degree to which VP2 enhanced infectivity was not as great as in 293TT cells. In contrast to the other cell lines, MCV infectivity of UACC-62 cells and SK-MEL-2 cells (both melanoma lines) was nearly unchanged when VP2 was present at any level, suggesting that VP2 is dispensable for MCV infectious entry in some cell types.

**Figure 5:**
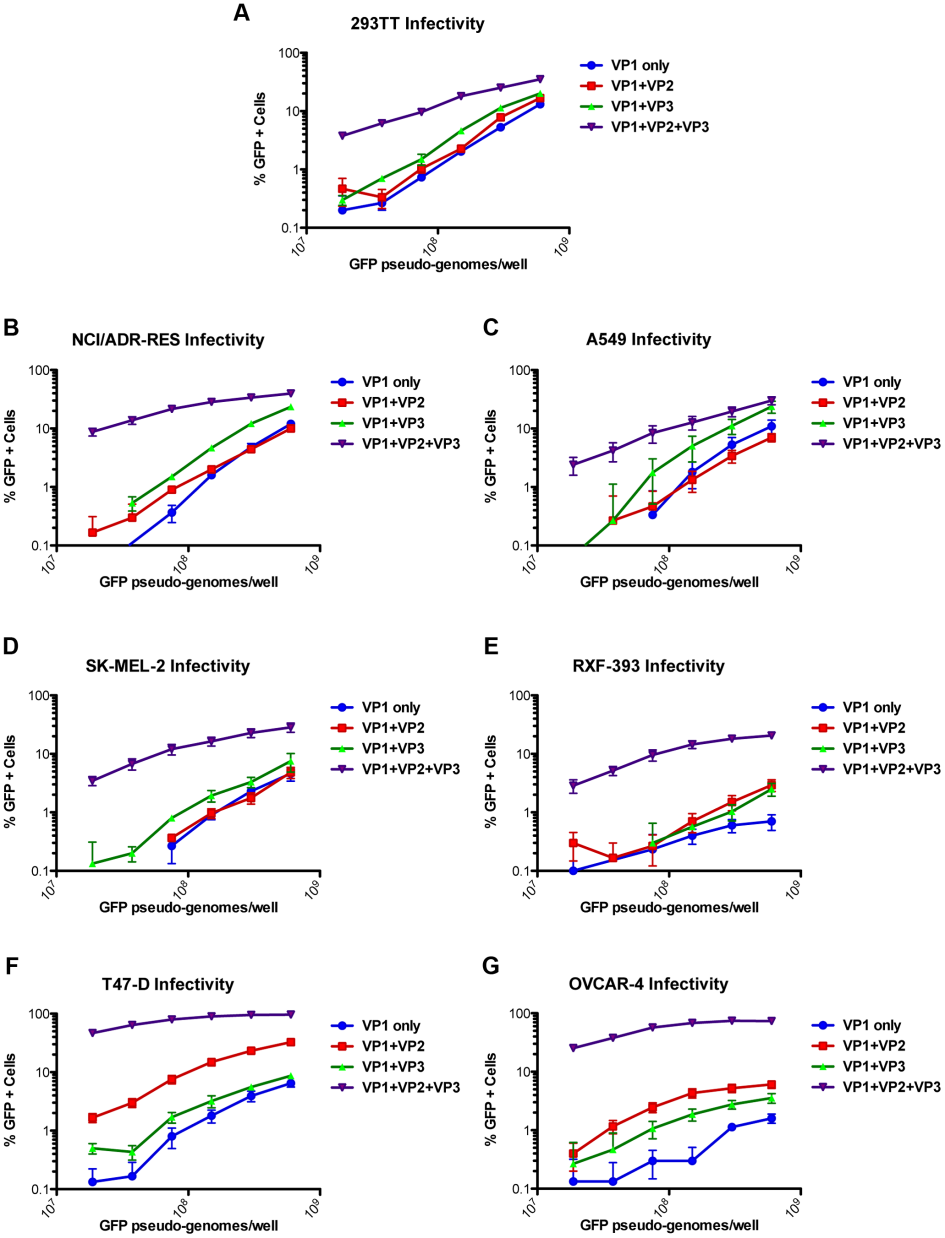
Effect of the VP2 and VP3 proteins of BKV on pseudovirus transduction. Transduction of different cell lines (A–G) by BKV pseudoviruses produced with the gene for VP1-only, VP1+VP2, VP1+VP3, or VP1+VP2+VP3 was analyzed by flow cytometry. The average percentage of GFP-positive cells from three experiments is shown and error bars represent the standard error of the mean.

To determine whether minor capsid protein-independent entry in some cell lines is a feature unique to MCV, we tested the VP2/VP3 dependence of another polyomavirus pseudovirus. BK polyomavirus (BKV) genotype IV pseudovirion transduction in multiple cell lines has also previously been established [42]. BKV pseudovirions were produced with VP1 alone, VP1+VP2, VP1+VP3, or VP1+VP2+VP3. Infectivity of the BKV pseudovirions was then examined in seven different cell lines, four of which were previously challenged with MCV pseudovirions (Figure 5A–D). The other three cell lines were chosen because they are highly BKV-transducible and represent diverse tissue types (Figure 5E–G). The infectivity of the BKV VP1-only pseudovirions was dramatically lower than the VP1+VP2+VP3 pseudovirions on all tested cell lines (Figure 5A–G). This analysis included the SK-MEL-2 cell line that MCV pseudovirions transduced in a VP2-independent fashion (Figure 4E and 5D). Thus, infectious entry of the BKV pseudovirus appears to differ from the MCV pseudovirus with regard to its dependence on the presence of minor capsid proteins.

**Figure 6:**
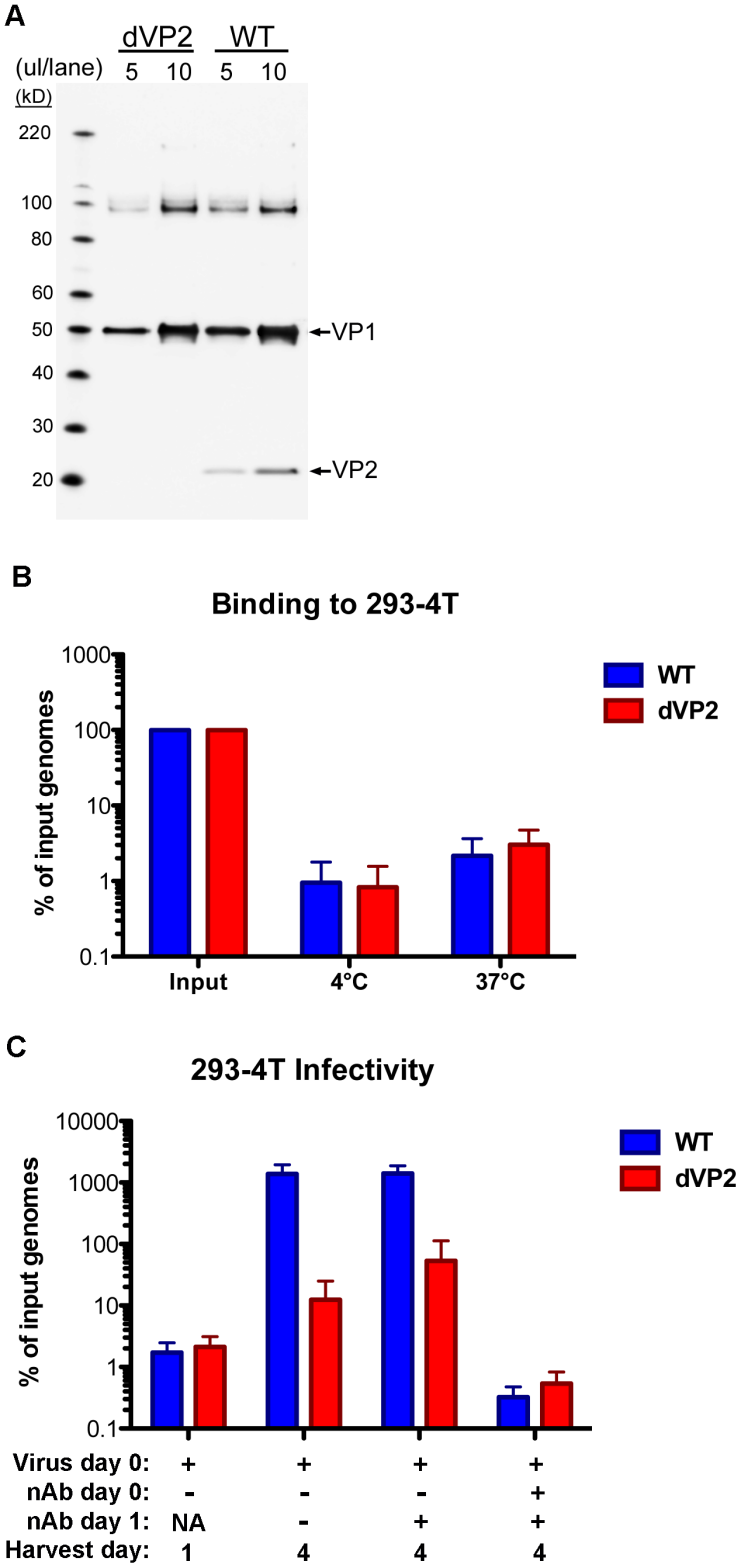
VP2-knockout in native MCV virions. (A) Purified WT or VP2-knockout mutant (dVP2) MCV virions were analyzed by SDS-PAGE and western blot with a mixture of anti-VP1 and anti-VP2 rabbit sera. (B) Equal amounts of WT and dVP2 virions with equivalent MCV genomic DNA content, were inoculated onto 293-4T cells. Samples were placed at 4°C or 37°C for one hour, and then washed prior to analysis of cell-associated viral DNA as below. (C) Equal amounts of WT and dVP2 virions were inoculated onto 293-4T cells with either neutralizing polyclonal anti-MCV serum (nAb +) or rabbit pre-bleed (nAb −). One sample was harvested the next day (Harvest day 1) while other samples were re-plated +/− neutralizing serum, then harvested day 4 post-infection. The percentage of viral DNA in samples, relative to the amount added in the form of virions, was determined by qPCR. The average of five experiments is shown and error bars represent the standard deviation.

That a minor capsid protein would not contribute to MCV infection in some cells is highly unexpected, and it would be desirable to verify the results with native MCV virions. Unfortunately, few cell types replicate MCV DNA to an appreciable level [43], [44], and 293-4T cells are the only cell type demonstrated to replicate MCV genomic DNA delivered by MCV infection to detectable levels above background [42]. As 293TT cells are the parent of 293-4T cells, the presence of VP2 in virions would be expected to contribute to efficient native MCV infection of 293-4T cells. To test this prediction, MCV genomic DNA was mutated with a frame-shift mutation to prevent VP2 production, and additional changes were made to the downstream sequence to ensure VP3 would not instead be produced by translational reinitiation. The mutant (dVP2) and WT native virions were produced as before and purified. A western blot of the virion preparations shows that only the WT virus contains VP2 protein (Figure 6A). The yield of dVP2 virions was similar to WT, and the rate of viral genome encapsidation was nearly identical between the two viruses (data not shown). Binding of the dVP2 and WT viruses to 293-4T cells proved to be equivalent in qPCR-based measurements of cell-associated MCV genome copies after one hour of incubation at 4**°**C or 37**°**C (Figure 6B). Infectivity of the dVP2 mutant was determined similarly to the dVP3 mutants. Duplicate wells of 293-4T cells were collected one day and four days post-infection with WT or dVP2 virions. In addition, a negative control was performed in which anti-MCV neutralizing antibodies were added at the time of infection or the following day. The addition of neutralizing antibodies at the time of infection demonstrates the validity of the infection assay, while neutralization one day after an initial round of infection controls for the possibility of multiple rounds of infectious spread. Both the WT and dVP2 viruses displayed increases in MCV genome copy number between day one and day four (Figure 6C), suggesting both were capable of infecting 293-4T cells. However, the apparent infectivity of the dVP2 virus was much less than WT, with ∼100-fold fewer replicated MCV genomes on day four. These results confirm that the MCV VP2 protein has an important function during infectious entry of native MCV virions into 293-4T cells.

**Figure 7:**
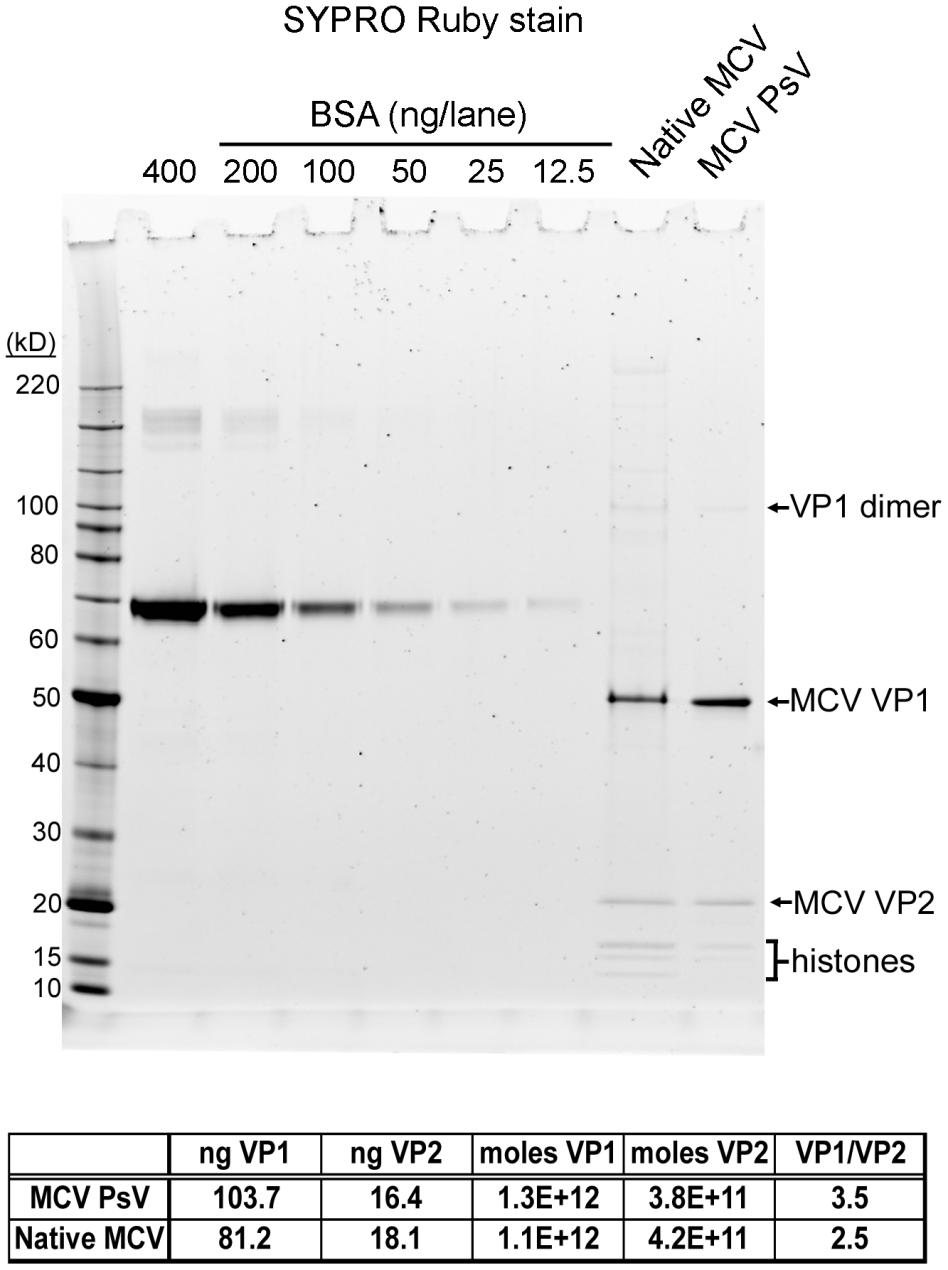
The ratio of VP1 to VP2 in MCV pseudovirions and native virions. BSA standards, MCV pseudovirions, and WT native MCV virions were analyzed by SDS-PAGE and SYPRO Ruby protein stain. A standard curve was created with density measurement of BSA standards and used to calculate estimates of VP1 and VP2 protein concentration. The table below the graph shows the result of these calculations.

Since the VP2 protein occupancy of MCV appears to be a strong determinant of infectious entry in multiple cell types, we carefully measured the apparent ratio of VP1∶VP2 in MCV pseudovirions and native virions. MCV pseudovirions with high VP2 content and WT native MCV virions were analyzed alongside BSA standards by SDS-PAGE and SYPRO Ruby stain (Figure 7). This protein stain is believed to show little protein-to-protein variability in staining intensity, as it binds primarily to the polypeptide backbone, with minor contributions from basic amino acid residues [45]. Since the MCV VP1 protein contains a higher percentage of basic residues than MCV VP2, the stain might be expected to slightly over-estimate the relative abundance of VP1. Quantitative analysis of the stained gels showed a VP1∶VP2 molar ratio of 5∶2 for native MCV virions (i.e., two molecules of VP2 per pentameric VP1 capsomer). MCV pseudovirions exhibited slightly lower VP2 occupancy, with a VP1∶VP2 ratio of 5∶1.4.

There are several possible roles that VP2 may be playing to promote the infectious entry of MCV into some cell lines. One possibility is that VP2 directs MCV through a cellular pathway that is suitable to accomplish infection, while particles lacking VP2 only rarely find this pathway. If this were true, it might be possible to visualize differences in subcellular localization of VP1-only and VP1+VP2 MCV particles by fluorescence confocal microscopy. Previously published studies found that EdU-labeling of human papillomavirus type 16 (HPV16) pseudovirion DNA is a sensitive method for the detection of virus-associated DNA throughout the entry process [46]. We therefore chose to label the DNA encapsidated within pseudovirions with EdU for high-specificity detection by Click-iT chemistry and imaging with co-localization of organelle markers. Since 293TT cells do not adhere well and do not have morphology suitable for microscopic analysis, the analyses were performed using the NCI/ADR-RES cell line, which supports VP2-dependent infectious entry (Figure 4C).

**Figure 8:**
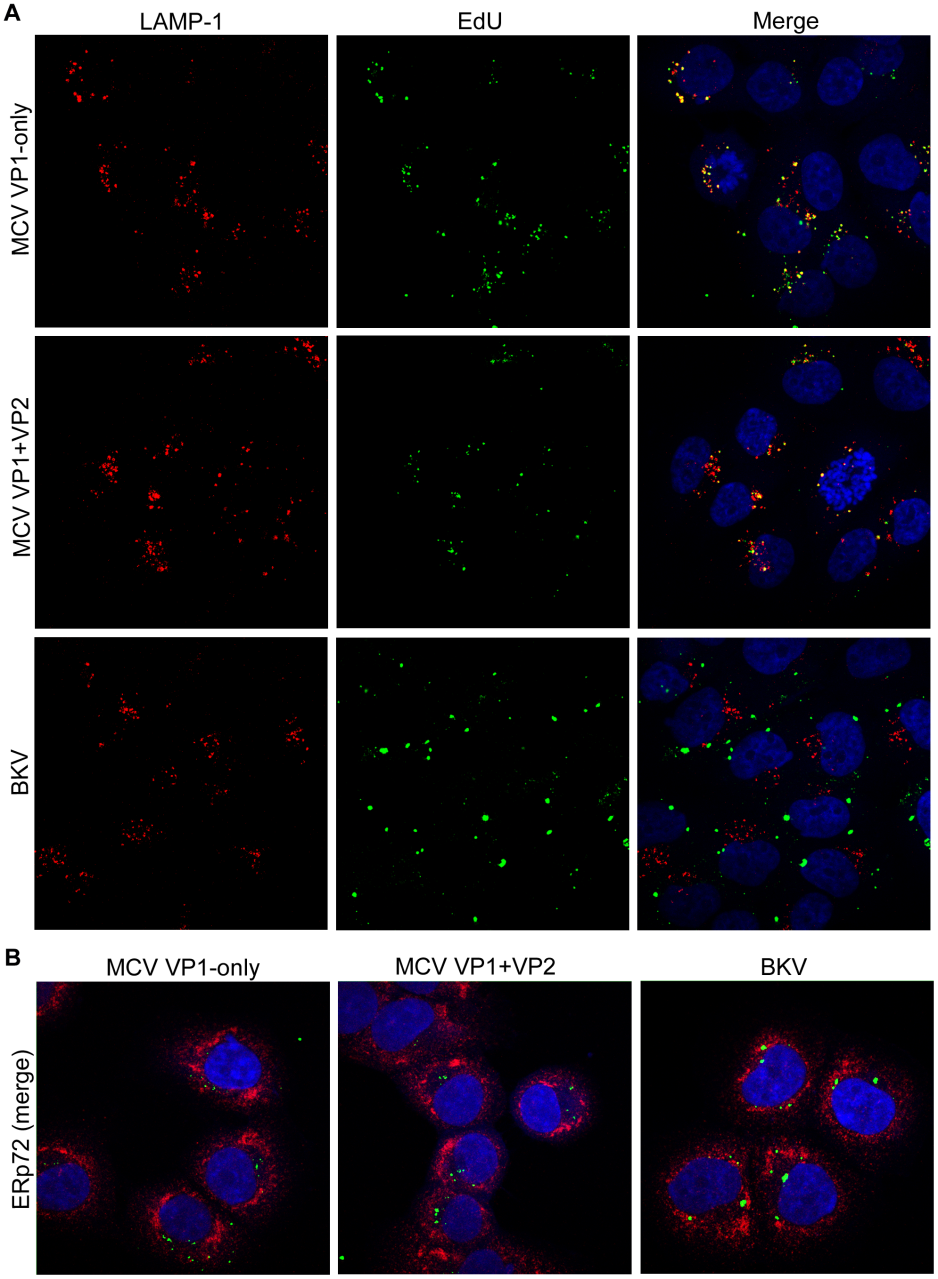
No discernible effect of VP2 on MCV trafficking. Purified MCV VP1-only, MCV VP1+VP2, or BKV pseudovirions produced with EdU-labeled encapsidated DNA were examined by confocal immunofluorescent microscopy in NCI/ADR-RES cells, 48 hours after pseudovirion inoculation. EdU was reacted with Alexa Fluor 488 (green), and then (A) LAMP-1-positive (late endosome/lysosome) or (B) ERp72-positive (ER) subcellular compartments were immunostained (red) and mounted with DAPI (blue).

MCV, with or without VP2, was visible in a punctate pattern within the cell by roughly six hours after pseudovirion inoculation (data not shown). This pattern changed little over a period of several days. The EdU signal did appear to increase significantly over time, likely resulting from increasing accumulation of particles in a single location (data not shown). Co-staining of EdU with LAMP-1 revealed strong co-localization of encapsidated DNA in the late endosome/lysosomal compartment (Figure 8A). The MCV VP1 protein also appeared to co-localize with LAMP-1+ compartments (data not shown). At no time was MCV convincingly co-localized with the ER markers calreticulin or ERp72, or the Golgi marker giantin, and EdU signal was rarely observed in the nucleus unless the nucleus was undergoing division (Figure 8B and data not shown). The presence or absence of VP2 did not discernibly alter these patterns of sub-cellular localization.

To address the concern that the cells might non-specifically sequester a majority of MCV particles in lysosomes for non-infectious degradation, BKV was also produced with EdU-labeling of DNA and examined alongside MCV. BKV was not found co-localized with the LAMP-1 marker, and the pattern of EdU detected in BKV-transduced NCI/ADR-RES cells clearly differed from MCV (Figure 8A). The majority of BKV particles amassed in a single location overtime, the identity of which was not revealed by any of our subcellular markers (Figure 8B and data not shown). The result shows that MCV's trafficking to LAMP-1+ compartments is a distinctive feature of MCV's biology.

MCV particles +/− VP2 were also examined by differential detergent subcellular fractionation [47], and, again, no effect of VP2 was detected using these methods (data not shown). The significance of these results is uncertain considering the particle-to-infectivity ratio of the polyomavirus pseudoviruses is such that it may be difficult to observe the small fraction of particles at a particular step of the productive infectious pathway at any given moment during the slow and potentially asynchronous process of entry into cells.

### Myristoylation of the MCV VP2 Protein

**Figure 9:**
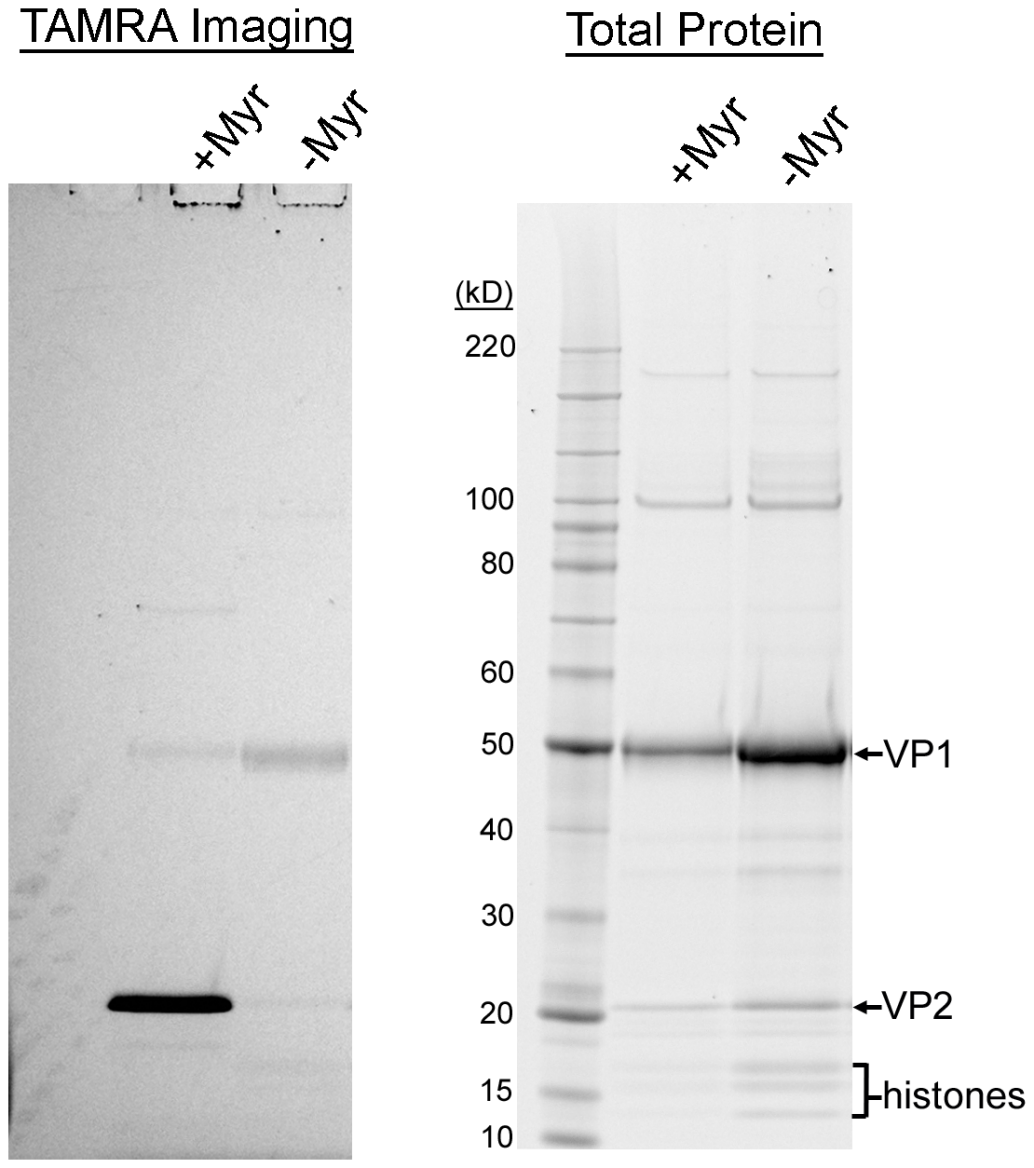
The MCV VP2 protein is myristoylated. MCV pseudovirions produced in the presence (+Myr) or absence (−Myr) of myristic acid-azide were reacted with TAMRA-alkyne. Proteins were precipitated and separated by SDS-PAGE for in-gel examination of TAMRA fluorescence. The same gel was then stained with SYPRO Ruby to detect all proteins.

The intriguing differences between MCV and previously studied polyomaviruses inspired further investigation of the VP2 protein. Whether the VP2 N-terminal consensus sequence for myristoylation resulted in the covalent attachment of a myristoyl group was next examined. Traditional methods for analyzing myristoylation use tritiated substrates, and detection is time-consuming and cumbersome. We chose to instead utilize a recently developed approach involving Click-iT chemistry. Cells transfected with VP1 and VP2 expression plasmids were metabolically labeled (or mock-labeled) with myristic acid derivatized with an azide group. Pseudovirions were extracted from the cells, and then reacted with a Tetramethylrhodamine (TAMRA) fluorochrome linked to an alkyne. The highly specific copper-catalyzed reaction between the azide and alkyne permits in-gel fluorescent detection of myristoylated proteins following SDS-PAGE. The same gel that is examined for TAMRA fluorescence can then be stained for total protein. The results clearly indicate that the MCV VP2 protein is myristoylated, as only VP2 of the metabolically labeled pseudovirus displayed significant TAMRA fluorescence (Figure 9).

**Figure 10:**
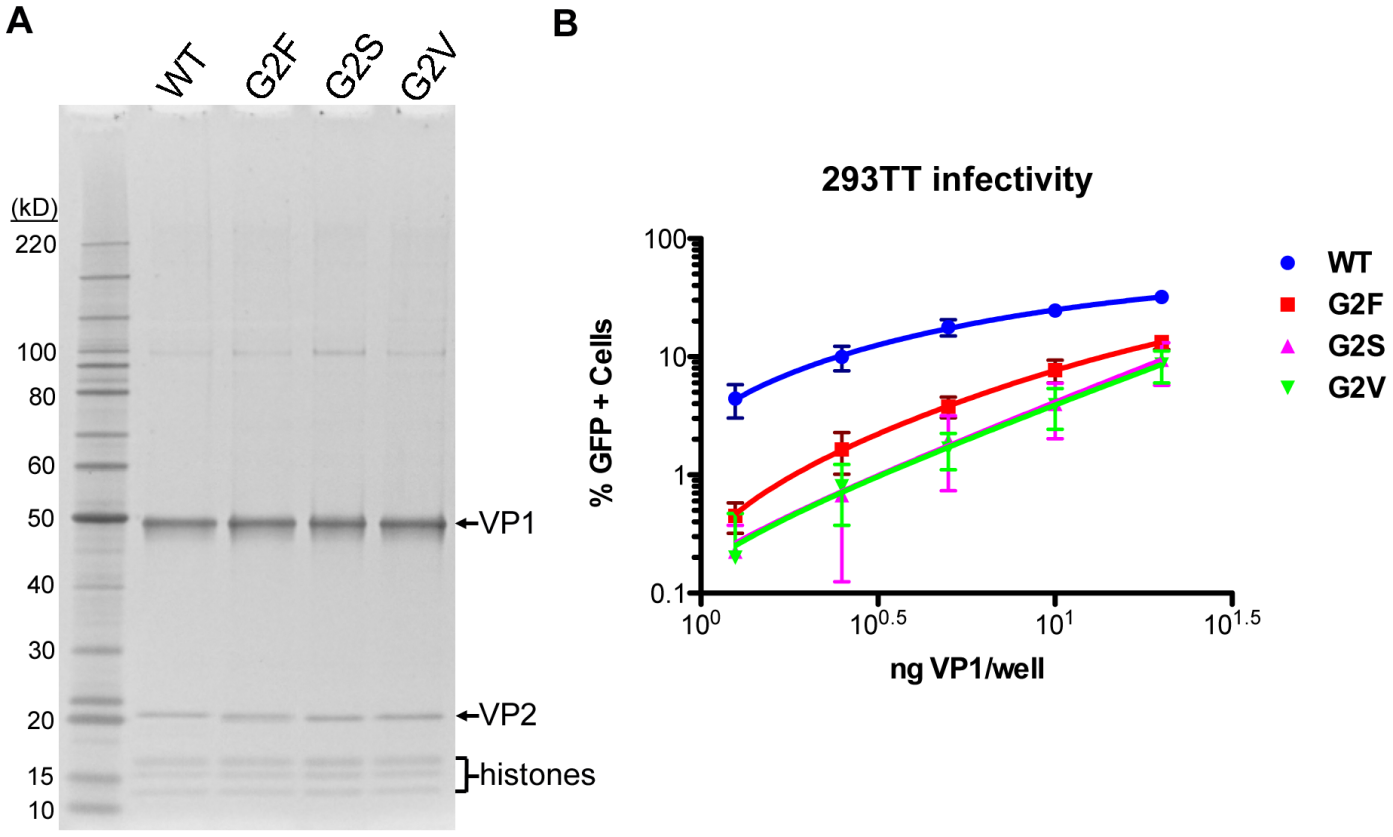
Effect of VP2 myristoylation on transduction. (A) Purified MCV pseudoviruses made with WT VP2 or Gly_2_-mutated VP2 (G2F, G2S, and G2V) were analyzed by SDS-PAGE and SYPRO Ruby protein stain. (B) The infectivity of MCV pseudoviruses containing WT VP2 or Gly_2_-mutated VP2 was determined in 293TT cells using flow cytometry. The average percentage of GFP-positive cells from four experiments is shown and error bars represent the standard deviation.

In previously studied polyomaviruses, the glycine at position two of the VP2 protein sequence is required for covalent attachment of a myristoyl group [10]. Mutation of MPyV VP2 Gly_2_ has previously been shown to reduce the infectivity of virions [9], [11]. We mutated the Gly_2_ of MCV to valine (G2V), serine (G2S), or phenylalanine (G2F). Pseudovirions were produced and purified with the mutated VP2, then examined by SDS-PAGE (Figure 10A). Infectivity of the mutant pseudoviruses was reduced by ∼10 fold on 293TT cells (Figure 10B). VP2-null MCV pseudovirions displayed a ∼20–30 fold decrease in 293TT cell transduction relative to high-VP2 occupancy pseudovirions. Thus, myristoylation is an important feature of the MCV VP2 protein.

### Phylogenetic Analyses of Apparently VP3-Less Polyomavirus Species

A large number of human and animal polyomavirus species have been discovered in recent years, and the family *Polyomaviridae* now includes about 65 known species. Inspection of VP2 protein sequences reveals that more than a quarter of known polyomavirus species lack the consensus VP3 N-terminal MALXXΦ motif (Table S1 and Figure S1 alignment position 170–185). For the purposes of phylogenetic analyses, we defined polyomavirus species with a clear homolog of the VP3 N-terminal motif as “VP3^+^” and species lacking the motif as “VP3-less.”

**Figure 11:**
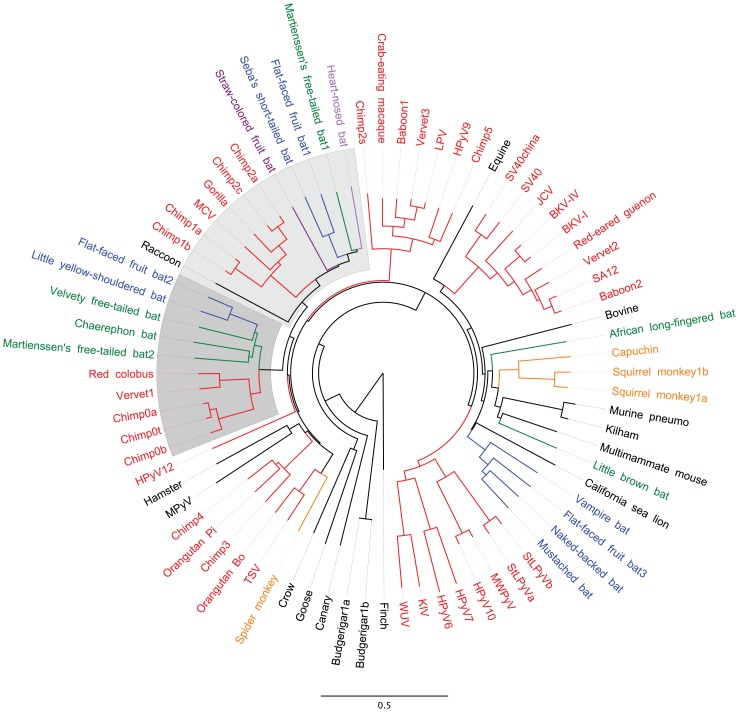
VP3-less polyomaviruses are a distinct monophyletic clade. A neighbor-joining tree was constructed based on a MUSCLE alignment of the complete genome sequences of representatives of known polyomavirus species. Species were assigned nicknames based on common English names of host organisms (for naming key see Table S1). Host species thought to have shared a common ancestor within the past 35 million years are assigned common colors. Host animal species shown in black were excluded from the color-coding scheme. The two lobes of the apparently VP3-less clade are shaded.

When mapped onto a phylogenetic tree drawn based on an alignment of the complete nucleotide sequences of polyomavirus genomes, VP3-less species cluster together into a discrete monophyletic clade (Figure 11). The VP3-less clade is sub-divided into two separate monophyletic lobes. Similar patterns were observed for VP3-less species in phylogenetic trees drawn based on alignments of VP1, VP2, and Large T antigen protein sequences (data not shown). A simple model for these results is that members of the VP3-less clade all descended from a single viral ancestor that was not shared with the VP3^+^ viruses. In other words, the loss of VP3 appears to have involved a single bottlenecked historical event, as opposed to having arisen repeatedly through convergent evolution.

The VP2 ORFs of VP3-less species are strikingly shorter than those of VP3^+^ species. VP3-less VP2 ORFs range from 229–243 codons, while VP3^+^ VP2 ORFs range from 304–416 codons (Figure S4). VP3-less species also tend to encode relatively long VP1 ORFs (435±44 codons in VP3-less species versus 372±22 codons for VP3^+^ species, with error representing one standard deviation). The shorter VP2 ORF length of the VP3-less viruses is largely attributable to a sharply defined deletion of a ∼50 amino acid segment within the C-terminal 1/3^rd^ of all VP3^+^ VP2 proteins (see Figure S1, alignment positions 300–390). Although the missing segment of VP2 has not previously been assigned any function, it includes the methionine codon that initiates translation of VP4, a cytolytic protein expressed by SV40 [48]. The deleted segment contains a conserved extended sequence motif that can be summarized as YxxLxxYYxxL(x)PxxPxxxR. There are no discernible homologs of the deleted VP2 segment in VP1 proteins (see Discussion). Intriguingly, the L2 minor capsid proteins of many papillomaviruses encode a motif (YYxxLxPxxP) similar to the core of the VP2 motif.

In addition to the ∼50 amino acid deletion, the VP3-less polyomaviruses, as well as a handful of VP3+ polyomavirus species, are missing an additional ∼30 amino acid segment at the C-terminus of VP2. This patch is highly basic, and a nuclear localization signal (NLS) has been identified in SV40 VP2/3 in this region [49]. In SV40, the VP2/3 NLS overlaps a proposed DNA-binding motif [50]. MPyV VP2/3 proteins appear to have a truncated C-terminus, consistent with the observation that nuclear localization of MPyV VP2/3 requires co-expression of VP1 [51]. Various polyomavirus VP1 and VP2 protein sequences were analyzed using an NLS-prediction algorithm, cNLS Mapper [52]. The algorithm correctly predicted the NLS phenotypes of SV40 and MPyV VP1 and VP2/3 proteins and predicted an MPyV-like phenotype for MCV, with a strong NLS motif predicted near the N-terminus of MCV VP1 and no predicted NLS within MCV VP2. Like MCV, all VP3-less polyomaviruses encode predicted NLS sites near the N-terminus of VP1 and no predicted NLS within VP2 (Table S1 and Figure S5). In contrast, nearly all VP3+ polyomaviruses carry predicted strong NLS sequences near the C-terminus of VP2 (with MPyV being a noteworthy exception). About half of VP3+ species do not encode a predicted NLS within VP1.

**Figure 12:**
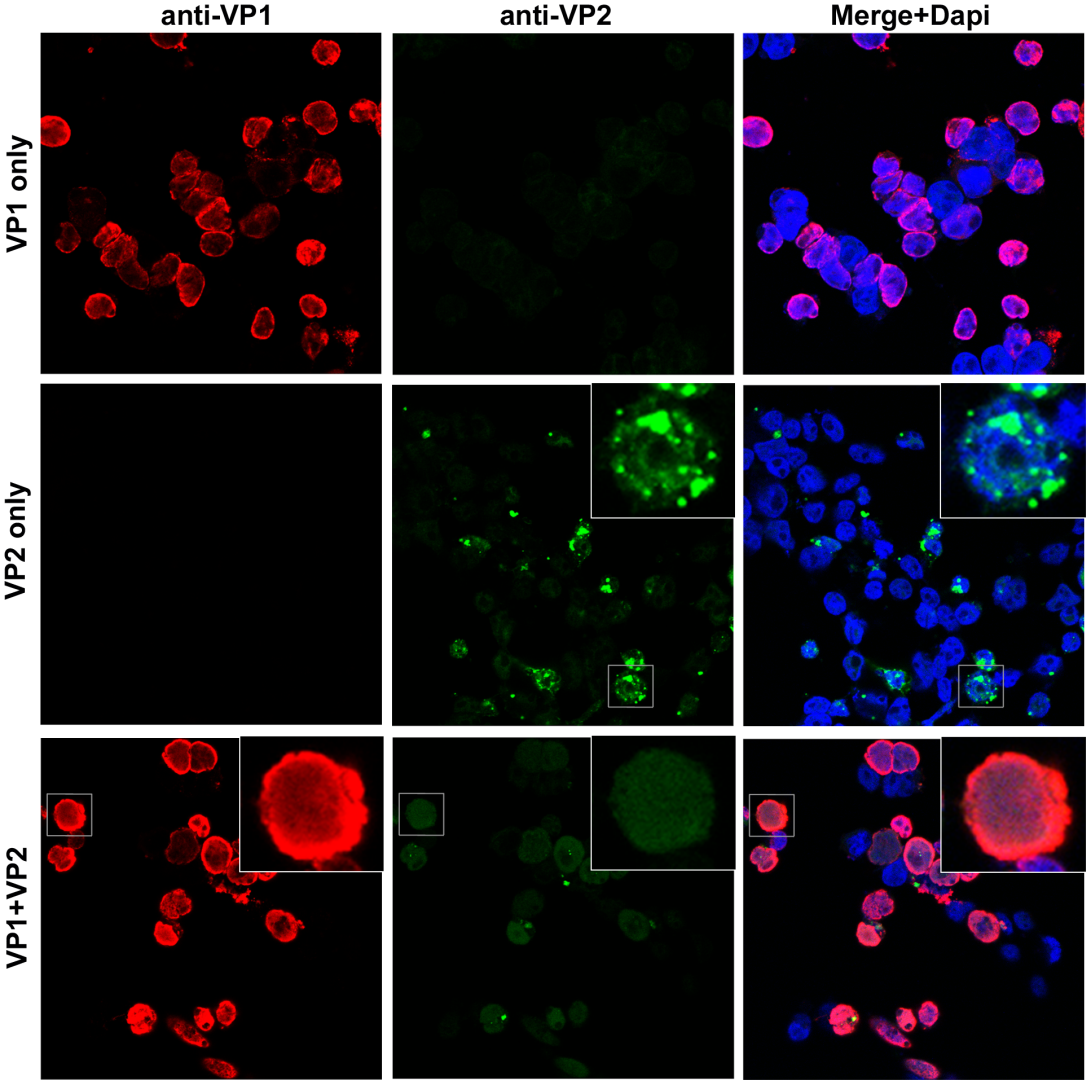
Nuclear localization of MCV VP2 expressed +/− VP1. Confocal immunofluorescent microscopy of 293TT cells transfected with VP1 (red), VP2 (green) or VP1 and VP2 from a bicistronic plasmid. A mouse monoclonal antibody was used to detect VP1, and a rabbit polyclonal antibody was used to detect VP2. Immunostained coverslips were mounted with DAPI (blue).

To confirm the predicted NLS phenotypes of MCV VP1 and VP2, we examined the subcellular localization of each protein using confocal microscopy in 293TT cells (Figure 12). MCV VP2 primarily appeared in large, peri-nuclear cytoplasmic punctae when expressed alone. In contrast, VP1 expressed alone showed a diffuse nuclear pattern with apparent enrichment around the interior rim of the nucleus. When VP1 and VP2 were co-expressed in 293TT cells, there was a clear shift in the majority of VP2 localization to a diffuse nuclear pattern.

## Discussion

MPyV, SV40, BKV, and JCV have been studied extensively in the roughly half-century since their discovery. MCV is very distantly related to these better-studied viruses and thus presents an important opportunity to learn which biological features are shared among all polyomaviruses and which features have diverged during the course of the family's evolution. At the time MCV was discovered, it was the only example of a polyomavirus with a VP2 protein lacking the consensus MALXXΦ motif that forms the N-terminus of known VP3 proteins. It was thus unclear whether MCV encoded an unusual VP3 protein or entirely lacked a VP3 protein. In this work, we show conclusively that MCV lacks a VP3 minor capsid protein. This feature of MCV appears to be typical of an emerging clade of polyomaviruses encompassing more than a quarter of currently known species.

Although high-sensitivity western blotting did not detect VP3 proteins in MCV virions or in MCV-infected cells it is difficult to absolutely rule out the possibility that VP3-like proteins might be expressed under some conditions in vivo or that VP3-like proteins might play a non-virion role in the viral life cycle. One example of a possible non-virion role is illustrated by the VP4 protein of SV40. VP4 is not incorporated into SV40 virions, but instead exhibits a cell-lytic activity that is important for virion release [48]. SV40 VP2, VP3, and VP4 proteins have all been shown to permeabilize membranes [53]. In contrast, we have not observed cytopathic or lytic effects in cell cultures replicating and producing native MCV virions, nor have others [41]–[44]. In addition, we observed no change in cell proliferation when VP2 or possible VP3 proteins were deleted from the replicating virus. Consistent with these observations, transient overexpression of individual VP2 or VP3 proteins in the presence or absence of VP1 does not appear to cause cytolytic effects in 293TT cells (data not shown). It thus seems unlikely that any products of the MCV minor capsid protein ORF could play an SV40 VP4-like role in the MCV life cycle. It is possible that MCV, like papillomaviruses, does not require an active lytic process for release. It is currently uncertain which cell types are productively infected by MCV, but the virus appears to reside in the skin, where the natural host process of keratinocyte desquamation might facilitate release of virions into the environment [21], [54], [55].

Most current literature states that polyomavirus virions contain an average of one minor capsid protein (either VP2 or VP3) per capsomer [56]. However, a careful review of older primary literature suggests that this established view is probably incorrect. An early report by Estes, Huang, and Pagano used radiolabeling and spectrophotometry to show that the molar ratio of VP1∶VP2∶VP3 in SV40 virions is 6∶1∶1.5 [57]. This ratio measurement implies a total of about two minor capsid protein molecules per pentameric VP1 capsomer. A later study by Lin and colleagues asserted, as data not shown, that SV40 virions have a capsid protein ratio of 16.88∶1∶2.65, indicating about one molecule of minor capsid protein per capsomer [58]. Lin and colleagues' conflicting claim was based on Coomassie-stained SDS-PAGE gels, raising the caveat that the ratio calculation may have been distorted by differential staining of VP1, VP2, or VP3 by the Coomassie dye. Our current analyses of native MCV virions using a less variable staining reagent appear to confirm Estes and colleagues' original biophysical results showing that native polyomavirus virions can accommodate an average of two molecules of minor capsid protein per VP1 capsomer.

Our findings show that MCV VP2 is required for efficient transduction of some cell types but not others. A model for this finding is that, in cells where VP2 is beneficial, there is a barrier to entry that VP2 helps overcome. During infection of cells such as UACC-62 or SK-MEL-2, which VP1-only pseudoviruses transduce efficiently, this barrier seems not to exist or is not encountered. Interestingly, all apparently VP3-less viruses encode larger VP1 proteins than those found in most putatively VP3^+^ species (Figure S4). We speculate that the larger VP1 proteins of the VP3-less viruses may have assumed some of the functions of the minor capsid proteins, thus making VP2 conditionally dispensable and rendering VP3 entirely dispensable. If so, then the expansion of VP1 would presumably have evolved prior to the internal VP2 deletion events that appear to have destroyed VP3 and truncated C-terminal portions of VP2.

MCV VP2 does not exhibit strong nuclear localization unless it is co-expressed with VP1 (Figure 12). Analyses using an NLS prediction algorithm suggest that, like MCV, all VP3-less polyomavirus species encode an NLS near the N-terminus of VP1 and do not encode an NLS within VP2. In contrast to MCV, nearly all VP3+ species have a predicted NLS near the C-terminus of VP2, and about half of these species do not contain a predicted NLS within VP1. Polyomavirus genomes are organized such that the coding regions for the N-terminus of VP1 and C-terminus of VP2 overlap (Table S1). It is thus possible to envision frameshift mutations or tandem duplication events that would result in the transfer of NLS motifs (and possible nearby DNA-binding motifs) from the C-terminus of VP2 to the N-terminus of VP1 (or vice versa). This might represent a concrete example of transfer of a VP2 function to VP1 during the evolution of VP3-less species. Intriguingly, several polyomavirus species show evidence of sequence duplication in or near the VP2/VP1 overlap region (noted in Table S1).

Polyomaviruses are believed to be constrained by the small size of their ∼5 kb genomes and it seems unlikely that unnecessary genes would be maintained over evolutionary timeframes. Thus, the fact that the VP2 gene is maintained in all known polyomavirus species suggests that VP2 plays an important role in MCV biology in vivo. However, it remains possible that the apparently VP2-independent infectious route is also relevant to pathobiology. A recent publication from Muñoz and colleagues documents an outbreak of hamster polyomavirus (HaPyV)-induced lymphoma [59]. Interestingly, most HaPyV genomes isolated from lymphoma cells were found to have deletion mutations encompassing the N-terminus of VP2 and, in one instance the N-terminus of VP3 as well. Although it is unclear whether these natural VP2/3 deletion mutations play a causal role in lymphoma development, our data open the possibility that loss of VP2 might lead to altered viral tropism, conceivably with pathogenic consequences.

It is not yet known if the cell types MCV productively infects in vivo are the same cells that MCV pathogenically transforms, nor whether the virus infects both classes of cells via the same entry mechanism. We have previously examined the transduction efficiency of MCV in four MCC lines and found each MCC line to be highly resistant to transduction with, at best, ∼2% of cells becoming transduced with maximum doses of MCV pseudovirus [42]. Although it may seem counter-intuitive that MCC lines carrying integrated MCV genomes are resistant to MCV infection, it is important to note that progression to malignancy and adaptation to culture are typically associated with a large number of genetic, epigenetic, and phenotypic changes [60]. This problem is illustrated by a current debate over whether MCC tumors arise from differentiated Merkel cells, epidermal stem cells, or B cells [61], [62]. To partially address the problem that cultured cell lines may not faithfully represent their tissue of presumed origin, we examined an assortment of different cell lines that MCV readily transduces. Given the abundance of MCV in human skin, it is intriguing that the two cell lines that MCV VP1-only particles transduced efficiently were derived from skin tumors (melanoma). However, further analysis would be required to determine if this reflects a common feature of primary melanocytes in vivo or is simply a coincidence.

Initially, we hypothesized that VP2 may direct the trafficking of MCV down a productive infectious entry pathway, but our data suggest that the MCV VP2 protein does not alter the bulk trafficking of particles. VP2 also does not affect packaging of viral DNA nor does VP2 affect the binding of particles to cell surfaces. Thus, it appears that VP2 functions at a step late in entry. Given our observation that myristoylation of VP2 is important for its function during entry, it is tempting to postulate that this hydrophobic modification is important for disruption of cellular membranes during entry. However, our microscopy data are equally consistent with other possible functions of the MCV VP2 protein during the late phase of infectious entry.

A controversial question in the taxonomy of the viral family *Polyomaviridae* is whether polyomavirus species usually arise through co-evolution with a particular host animal lineage, or whether individual polyomavirus species have sometimes evolved after being productively transmitted between distantly related animal families [17], [63]. Finding highly similar polyomavirus sequences in distantly related animal families would constitute strong evidence for transmission between animal families. Our phylogenetic analysis shows that the VP3-less clade can be divided into two separate lobes (Figure 11). Within each lobe, the phylogenetic relationships of individual polyomavirus species roughly resemble the phylogenetic relationships of the host animal families in which the viruses were discovered. Most notably, there are no examples of bat-derived viruses co-occupying short branches with primate-derived viruses. A simple model for the observed phylogenetic relationships would be that two distinct VP3-less polyomavirus species both infected the most recent common ancestor of placental mammals (or its near relatives), and the two VP3-less lineages evolved within different host animal families during the ensuing ∼100 million years. Such a languid evolutionary pace is consistent with molecular clock estimates previously proposed for papillomaviruses, which also have a small circular dsDNA genome that is replicated by host cell polymerases [64].

Taken together, the results clearly indicate that the MCV VP2 gene typifies a large clade of polyomaviruses that differ significantly from previously examined polyomaviruses, in that they do not encode a VP3 protein. Awareness of this fundamental difference between MCV and other polyomaviruses will be important for future studies aiming to understand the pathobiology of MCV and may guide efforts to prevent this extremely common and occasionally pathogenic infection.

## Materials and Methods

### Cells

293TT cells [65] were maintained in DMEM (Mediatech, Inc.) with 10% fetal bovine serum (FBS, Sigma), Glutamax-I (Invitrogen) and MEM non-essential amino acids (Invitrogen) supplemented with hygromycin B (250 µg/ml; Roche). 293-4T cells [41] were maintained in the same medium as 293TT, except supplemented with zeocin (100 µg/ml; Invitrogen) and blasticidin S (5 µg/ml; Invitrogen) rather than hygromycin. NCI/ADR-RES cells, A549 cells, SK-MEL-2 cells, UACC-62 cells, RXF 393 cells, OVCAR-4 cells, and T-47D cells were obtained from the Developmental Therapeutics Program (NCI/NIH). These lines were cultured in RPMI 1640 medium (Mediatech, Inc.) supplemented with 5% FBS and 1% Glutamax-I.

### VP2 Antiserum

Anti-VP2 polyclonal serum was raised by immunizing a rabbit (Lampire Biological Products) with purified recombinant MCV VP2 immunogens expressed in bacteria. The rabbit was initially primed with a maltose binding protein (MBP)-VP2 fusion protein expressed from plasmid pMVP2M, which was made by PCR-mediated transfer of the VP2 ORF of ph2m [15] into the HindIII and BamHI sites of pMXB10 (NEB). The fusion protein was expressed in T7 Express lysY/Iq *E. coli* (NEB) and purified over amylose resin according to the manufacturer's instructions. The VP2 PCR product incorporated a recognition site for tobacco etch virus (TEV) protease between the MBP and VP2. The purified fusion protein was cleaved using an improved TEV protease variant, S219P, expressed from plasmid pRK792 [66], which was a generous gift from David Waugh (NCI). The rabbit was primed with the MBP-VP2 immunogen in complete Freund's adjuvant, boosted once with MBP-VP2 in incomplete Freund's adjuvant, then given a final boost with a 6× histidine (His)-tagged VP2 protein expressed in bacteria using plasmid pHisMVP2. This plasmid was generated by transferring an EagI/NheI fragment of ph2m into pProEXhta (Invitrogen) cut with EagI and XbaI. The His-tagged booster VP2 immunogen was not treated with TEV protease. The final hyper-immune serum did not show any detectable neutralizing activity against MCV reporter pseudovirions (data not shown).

To validate the VP2 antiserum, we generated MCV pseudovirion standards by co-transfecting 293TT cells with plasmids containing codon-modified versions of the MCV VP1 and VP2 genes. We also produced pseudovirions using a separate VP3-only expression plasmid. Comparison of the VP2 and VP3 pseudovirus standards in SYPRO Ruby stained gels (data not shown) and western blots showed that the VP2 antiserum recognizes both VP2 and VP3 with similar efficiency.

### Native MCV Virion Production

MCV virions were produced using previously described methods [42], with slight variations. Briefly, 2.5 million 293TT cells were plated in a 25 cm^2^ flask the day prior to transfection. The cells were co-transfected with 5 µg re-ligated MCV isolate R17b (GenBank accession number HM011556.1) genomic DNA as well as 3.5 µg of MCV small t antigen (pMtB) and 4 µg of MCV large T antigen (pADL*) expression plasmids. Transfected cells were expanded for 5–6 days, and virions were harvested and purified over Optiprep ultracentrifuge gradients as previously described. For propagative amplification of native MCV virions, 293-4T cells were infected with these native MCV virions produced by transfection. Propagated virions were harvested from a portion of the infected culture every few days beginning on day 10 after initial virion inoculation. Infected cells were lysed, clarified, and purified over Optiprep ultracentrifuge gradients as previously reported [41].

Quantification of encapsidated viral DNA in WT and mutant native virions was determined by first digesting 5 µl of viral capsids from each purified preparation with 20 mM Tris, pH 8.3, 20 mM DTT, 20 mM EDTA, 0.5% SDS, and 0.2% proteinase K for 20 min at 50**°**C. DNA was then purified out of the digest using the QIAquick PCR purification kit (QIAGEN), and qPCR was performed with primers 5′-GCTTGTTAAAGGAGGAGTGG-3′ and 5′-GATCTGGAGATGATCCCTTTG-3′ to quantitate viral genomic DNA using the DyNAmo SYBR Green qPCR kit (NEB). Comparison to pMCV-R17a DNA standards permitted calculation of the concentration of viral DNA in each virus stock.

Mutation of MCV isolate R17b genomic DNA to create the dVP2 and dVP3(a/b/d) mutants, was accomplished using overlap PCR. PCR products were transferred into the pMCV-R17b vector using AvrII and BsrDI restriction site cloning.

### Virion Pull-Down and Western Blot

Approximately 400 µl of Optiprep-purified virions from a mixture of day 13 and day 17 post-infection harvests were immunoprecipitated with the anti-VP1 monoclonal antibody MV23 [67], which had been pre-complexed with protein G Dynabeads (Invitrogen). The sample was then eluted in NuPAGE LDS sample buffer (Invitrogen) with 40 mM DTT and electrophoresed through a NuPAGE Novex 4–12% bis-tris gel alongside the VP2 and VP3 standards described below. The gel was transferred to nitrocellulose and western blotted using rabbit VP2 antiserum diluted 1∶100.

### Native Virus Binding and Replication

The ratio of VP1 to packaged viral DNA was nearly identical in WT and dVP2 viral preparations, such that an equal concentration of VP1 (0.05 ng virus/well, ∼2.4×10^8^ copies) was added to 2×10^5^ 293-4T cells that were brought into suspension by trypsin treatment. To measure cell binding by the WT and mutant virus, each was incubated with cells for one hour at either 4**°**C or 37**°**C in an untreated, round-bottom 96 well plate. Cells were then washed three times before freezing in modified Hirt buffer I [68]. To examine dVP2 infectivity, WT or dVP2 virions were added in quadruplicate to cells that were just plated in a 24-well plate. As a control, neutralizing rabbit polyclonal antibody raised against MCV capsids was added to one of the quadruplicate wells, while pre-immunization serum was added to the other three. Roughly 24 hours later, all wells were incubated in trypsin to resuspend the cells, the trypsin was neutralized with growth medium, and cells were pelleted. Medium was changed in the sample containing neutralized virus and in two non-neutralized samples, and cells were re-plated in a larger well for continued growth. At this time, neutralizing serum was replaced in the population that had received it previously. Additionally, neutralizing serum was added to another infected population to prevent further spread of the virus. One infected cell population remained exposed only to pre-immunization serum. The fourth sample (Day 1) was resuspended in Hirt buffer I, then frozen. A total of four days after virus inoculation, the re-plated cells were collected by trypsinization and resuspended in Hirt buffer I. Low molecular weight DNA from all samples was isolated by modified Hirt extraction [68], and the number of genomic copies of MCV in the samples was determined by quantitative PCR as described previously [41].

Measurements of dVP3(a/b/d) infection were performed similarly to dVP2, but without the added neutralization controls. A volume of virus equaling 2×10^6^ copies of genomic DNA was added to 2×10^5^ 293-4T cells that were just plated in duplicate. The next day, cells were resuspended, pelleted and then either resuspended in Hirt buffer I and frozen, or resuspended in growth medium and re-plated for continued growth. On the fourth day after virus inoculation, the final set of cells was collected and all samples were subjected to modified Hirt extraction and MCV genome copies were measured by qPCR.

### Pseudovirus Production and Purification

MCV reporter pseudoviruses were produced using methods reported previously [15], [41]. In brief, 293TT cells [65] were transfected with plasmids expressing codon-modified versions of the major and minor capsid genes, as described below. Pseudoviruses carrying GFP reporter plasmids were co-transfected with pYafw [65] and/or pEGFP-N1 (Clontech). These plasmids express the GFP reporter under control of recombinant human elongation factor 1**α** promoter or cytomegalovirus (CMV) immediate early promoter, respectively. Separate pseudovirus stocks carried a mixture of reporter plasmids carrying a *Gaussia* luciferase reporter gene under control of CMV promoter (pCLG) or human elongation factor 1**α**(pLGluc). Except where indicated, transfected cells were harvested forty-eight hours after transfection. Cells were lysed in PBS supplemented with 0.5% Triton X-100 as well as Benzonase (Sigma) and Plasmid Safe (Epicentre) nucleases. Pseudovirions were purified over Optiprep gradients according to previously reported methods.

The pseudoviruses containing WT or mutated VP2 proteins that were analyzed in Figure 3 were not Optiprep purified, but the cell lysates containing matured pseudovirions were clarified by centrifugation at 5,000×g. A different detergent, Brij 58 (0.35%; Sigma), which is less cytotoxic than Triton X-100, was used to lyse the cells at the time of harvest in order to preserve the health of target cells during transduction experiments.

The mutation of M46 in the VP2 protein was accomplished by PCR with ph2m [16] as a template. The XhoI restriction site used in cloning fell near enough to the mutation site to be incorporated into the primer. Degeneracy was engineered into the primer and multiple clones were sequenced in order to obtain the various changes at this site. Pseudovirions were produced by transfection of 293TT cells with individual M46 mutants or the WT ph2m plasmid with pwM [16] and the pEGFP-N1 reporter plasmid.

Mutation of VP2 Gly_2_ was also accomplished with PCR using ph2m as the template and incorporation of a cloning restriction site (SnaBI) in the primer used for mutagenesis. Again, a degenerate primer was used to obtain multiple changes at the mutation site. In addition, the sequence upstream of VP2 was modified to improve the Kozak sequence. A sister construct, phK2m, in which the Kozak sequence was improved upstream of the WT VP2 gene, was also made. The primer used for construction of the myristoylation mutants is as follows: 5′-TTTTTTACGTAATATTGCCGCCACCATGKYNGGGATCATTACCCTGCTCGC-3′. Pseudovirions were produced by transfection of 293TT cells with individual Gly_2_ mutants or the WT ph2m plasmid with pwM and the pEGFP-N1 reporter plasmid.

MCV pseudoviruses with varying levels of VP2 incorporated were produced by transfection of pwM (VP1 only), pwM2m [67] (low VP2), or pwM2m plus ph2m (high VP2). In addition, the pLGluc and pCLG reporter plasmids were co-transfected with each of the MCV capsid protein plasmids. The pwM and ph2m plasmids, as well as both *Gaussia* luciferase plasmids, have within their plasmid backbones the gene for EGFP under the control of the SV40 promoter. The ph2m and pwM plasmids may be packaged by the pseudovirus particles in addition to the intended reporter plasmids, although pwM is slightly too large (6.6 kb) for efficient packaging. Since the number of luciferase and EGFP reporter gene copies relative to VP1 potentially differs between these pseudovirus preparations, the concentration of both reporter genes in each pseudovirus preparation was quantified by qPCR. To do this, the concentration of VP1 was first determined by densitometry of SYPRO Ruby-stained SDS polyacrylamide gels. DNA was then extracted from 100 ng of VP1 and analyzed by qPCR as above using primers specific for the reporter genes [41]. Comparison to plasmid standards permitted calculation of the concentration of reporter plasmid in each virus stock.

BKV pseudovirions were produced with the pLGluc and pCLG reporter plasmids and analyzed similarly to the MCV pseudovirions with varying VP2 levels. In addition to the reporter plasmids, BKV VP1-only pseudovirion production used the pwB plasmid, BKV VP1+VP2 used pwB2b and ph2b, BKV VP1+VP3 used pwB3b and ph3b, and BKV VP1+VP2+VP3 used pwB2b, pwB3b, ph2b, and ph3b. Harvest of BKV from transfected cells was performed similarly to MCV, except that cell pellets are treated with neuraminidase. The yield of each pseudovirus was similar, and DNA was extracted from 2 µl of each preparation for qPCR analysis of reporter gene content.

The purified pseudovirions used as protein standards to verify reactivity of the VP2 antiserum with both possible minor capsid proteins in western blot were produced by co-transfecting 293TT cells with the plasmid pwM with pC2m (VP2 under control of a CMV promoter) and/or pC3m (VP3 under control of a CMV promoter). The concentration of VP2 and VP3 in the pseudovirus stocks was first determined by densitometry of SYPRO Ruby-stained SDS polyacrylamide gels with comparison to BSA standards (data not shown).

Maps of plasmids used in this work and detailed virus production methods are available from our lab website http://home.ccr.cancer.gov/Lco/

### Cell Transduction Experiments

Cells transduced with MCV or BKV pseudoviruses were plated the day prior at the following concentration in a 96-well plate: 293-TT = 1.5×10^4^, A549 = 7.0×10^3^, SK-MEL-2 = 1.0×10^4^, UACC-62 = 5.0×10^3^, NCI/ADR-RES = 1.0×10^4^, RXF 393 = 7.0×10^3^, OVCAR-4 = 7.0×10^3^, T-47D = 1.0×10^4^. Cells were subconfluent at the time of inoculation, and transduced cells were incubated for three days prior to analysis. A two-fold dilution series of each virus stock was analyzed and, within each experiment, virus stocks were normalized to each other by VP1 concentration or reporter gene content. The top dose of VP1/well ranged from 20–50 ng, and VP1 standardization was used in all but the analysis of varying levels of MCV VP2 in pseudoviruses and the BKV +/− VP2 and VP3 pseudovirus infection. As indicated above, BKV pseudovirions and the MCV pseudovirions made with no, low, or high levels of VP2 were normalized by reporter gene content. The top dose of GFP reporter gene used was 3.0×10^8^ copies/well for MCV and 6.0×10^8^ copies/well for BKV. To measure pseudovirus-mediated transduction of the GFP gene, adherent cells were incubated with trypsin to detach them from the plate, transferred to an untreated 96-well plate and suspended in wash medium (WM; DPBS with 1% FBS, antibiotic-antimycotic, and 10 mM HEPES, pH 8). Cells were then analyzed by flow cytometry for GFP expression in a FACS Canto II with HTS (BD Biosciences).

### Cell Viability/Toxicity Analysis

Cell Proliferation Reagent WST-1 (Roche Applied Science) was added to the medium of 293-4T transfected with MCV isolate R17b genomic DNA, dVP2, dVP3(a/b/d), or pMtBS (MCV small t antigen). We have previously observed the toxic effects of transient MCV small t antigen over-expression [41], [69], so pMtBS was used as a positive control. Cells were transfected in a 24 well plate and split the following day into a 96 well plate in triplicate and a new 24 well. Five days later, WST-1 was added to the 96 well plate and absorbance was measured at several time points from 30 minutes to 3 hours. The cells in the 24 well plate were lysed and analyzed by SDS-PAGE and western blot for MCV VP1 (1∶8,000 dilution of rabbit VP1 antiserum) or MCV VP2 (1∶100 dilution of rabbit VP2 antiserum).

### EdU-Labeling and Detection

MCV pseudovirions containing only VP1 or VP1+VP2 were produced by transfection of 293TT cells with pwM or pwM2m and ph2m, respectively. The plasmids pIaw [70], ph2b and ph3b [41] were used to produce BKV pseudovirions. The pLGluc reporter plasmid was also included in each transfection mixture. Six hours after transfection, the medium of each culture was replaced with fresh medium containing 50 µM EdU-alkyne (Click-iT EdU Imaging Kit, Molecular Probes/Invitrogen). MCV pseudovirions were harvested ∼48 hours after transfection and purified normally using the methods described above. BKV pseudovirion harvest has been published previously and differs from MCV harvest by the addition of neuraminidase V during maturation [70]. The VP1 concentration of each pseudovirus preparation was determined by SYPRO Ruby stain of an SDS-polyacrylamide gel and densitometry with comparison to BSA standards.

For fluorescent imaging of EdU-labeled pseudovirions during cell entry, 3.5×10^4^ NCI/ADR-RES cells were plated on glass coverslips in a 24-well plate. The next day, MCV VP1-only or MCV VP1+VP2 pseudovirions equaling 20 ng of VP1/well or BKV pseudovirions equaling 40 ng VP1/well were inoculated onto the cells. After ∼48 hours, the cells were fixed with 2% paraformaldehyde and labeled with Alexa Fluor 488-azide using the Click-iT EdU Imaging Kit (Molecular Probes) according to the manufacturer's instructions. Co-staining was performed with an anti-LAMP-1 mAb (H4A3; Developmental Studies Hybridoma Bank) diluted 1∶300 or anti-ERp72 rabbit polyclonal antibody (Stressgen) diluted 1∶300. Alexa Fluor-488 conjugated MCV VLPs [67] were used to examine VP1 localization. Coverslips were inverted and mounted with Prolong Gold (Molecular Probes) containing DAPI. Images were acquired with a Zeiss LSM 780 confocal system interfaced with a Zeiss Axio Observer microscope. Images were collated with Adobe Photoshop Elements software, where the red levels of Figure 6A and green levels of Figure 6B were adjusted equally among images.

### VP1 and VP2 Localization

293TT cells were transfected with plasmids encoding MCV VP1 (pcM), MCV VP2 (p2mw) or MCV VP1 and VP2 (pMmw) were plated on poly-D-lysine/laminin coated coverslips. They were fixed with 2% paraformaldehyde and incubated in 0.1% Brij 58 in PBS with rabbit VP2 antiserum (1∶200) and mouse monoclonal MV16 hybridoma supernatant (1∶10). Alexa fluor (AF) secondary antibodies were used for VP1 (AF-594) and VP2 (AF-488) detection. Coverslips with mounted with Prolong Gold containing DAPI. Images were acquired with a Zeiss LSM 710 NLO confocal microscope and collated with Adobe Photoshop Elements software.

### Detection of MCV Myristoylation

Detection of myristoylation was performed by producing pseudovirions in cell growth medium supplemented with Click-iT myristic acid-azide (Molecular Probes) to a final concentration of 25 µM. Metabolic labeling was allowed to proceed for ten hours, and then pseudovirions were harvested using standard methods. The concentration of VP1 in this pseudovirus preparation and an unlabeled MCV pseudovirus preparation was determined by densitometry of a SYPRO Ruby-stained SDS-PAGE gel. An amount of each pseudovirion preparation equaling 1.9 µg of VP1, was mixed with 42 mM Tris, pH 8 and 0.83% SDS in a final volume of 60 µl. Tetramethylrhodamine (TAMRA)-alkyne was then reacted with the samples according to the manufacturer's instructions using the Click-iT Protein Reaction Buffer Kit. Proteins were then precipitated by methanol/chloroform extraction, and then resuspended in NuPAGE LDS sample buffer with 40 mM DTT. Proteins were separated by SDS-PAGE of the samples, and the gel was imaged under green epi-illumination in an ImageQuant LAS 4000 (GE Healthcare). The same gel was then SYPRO Ruby stained and imaged under epi-illumination with blue light.

### Viral Sequence Analyses

MacVector version 12.6 software was used to perform a MUSCLE alignment [71] on the nucleotide sequences of complete polyomavirus genomes. For display, each polyomavirus species was assigned a nickname based on the common English name for the animal host in which the virus was discovered or based on commonly used polyomavirus name abbreviations. A naming key, including accession numbers, is provided in Table S1. Curated compilations of sequences used for the analyses can be found at the following link: <http://home.ccr.cancer.gov/Lco/PyVE.asp>.

NLS prediction was performed using the following website: <http://nls-mapper.iab.keio.ac.jp/cgi-bin/NLS_Mapper_form.cgi> [52]. Bipartite NLS searching was restricted to terminal 60-amino-acid regions. For VP1, sequences with any NLS score of 4.5 or greater were considered NLS positive. In nearly all instances, the predicted VP1 NLS involved an N-terminal portion of the protein. Two exceptions were JCV and California sea lion VP1 proteins, for which the algorithm predicted only an internal NLS. The predicted JCV VP1 NLS, 282-QLRKRRVKN-291, is poorly surface-exposed in the crystal structure of the protein [72], consistent with the poor nuclear localization of JCV VP1 [73]. For VP2, sequences with a monopartite NLS score of greater than 3 were considered NLS-positive.

A neighbor-joining tree was constructed using random resolution of ties and Jukes-Cantor maximum likelihood method with proportional distribution of gaps. The tree was arbitrarily rooted on finch polyomavirus. Midpoint rooting or rooting on other Avipolyomaviruses or Wukipolyomaviruses also resulted in the appearance a monophyletic VP3-less clade, with similar relationships among members within the clade. For the tree shown in Figure 9, the root of the VP3-less clade and the two VP3-less sub-lobes each had bootstrap values of 100.

Assignment of the coloring scheme in Figure 9 was based on general comparisons between various sources, including Wikipedia and [74]–[76]. Greater weight was assigned to newer molecular phylogeny-based estimates than fossil record-based estimates.

## Supporting Information

Figure S1 **Alignment of polyomavirus VP2 proteins.** A representative set of VP2 proteins from known polyomavirus species was aligned using MUSCLE with PAM200 matrix. The names of polyomavirus species that lack the conserved VP3 MALXXΦ N-terminus motif (position ∼180 of the alignment) are marked with an asterisk.
(PDF)

Figure S2 **Viral protein expression from MCV genomic DNA in 293-4T cells.** Whole cell lysates of 293-4T cells transfected six days prior with WT or mutant MCV genomic DNA were separated by SDS-PAGE and western blotted with rabbit polyclonal VP1 antiserum (left) or rabbit polyclonal VP2 antiserum (right). The VP2 blot reveals no VP3-like proteins in cell lysates.
(TIF)

Figure S3 **Viability of 293-4T cells transfected with MCV genomic DNA.** WST-1 reagent was added to 293-4T cells transfected six days prior with WT or mutant MCV genomic DNA or a MCV small t antigen (sT) expression construct known to be cytotoxic. Absorbance was measured and averaged at multiple time points after WST-1 addition in triplicate wells. The average of three experiments is shown and error bars represent the standard error of the mean.
(TIF)

Figure S4 **VP1 and VP2 ORF length.** The y-axis shows the length (in number of codons) of the VP1 and VP2 ORFs for various polyomavirus species. The names of putatively VP3-less species are marked with asterisks.
(TIF)

Figure S5 **Polyomavirus phylogenetic trees displaying predicted nuclear localization sequences in VP2 or VP1.** Polyomavirus species from the same neighbor-joining tree shown in Figure 12 are color-coded to indicate a predicted lack of NLS in VP2 (red) or presence of NLS in VP1 (blue).
(TIF)

Table S1 **Polyomavirus naming key and capsid gene characteristics.**
(XLSX)
