# Supplementary material for: The Merkel Cell Polyomavirus Minor Capsid Protein
Source: PLoS Pathog. 2013 Aug 22;9(8):e1003558. doi: 10.1371/journal.ppat.1003558 (PMC3749969; doi:10.1371/journal.ppat.1003558)

Formatted Alignments

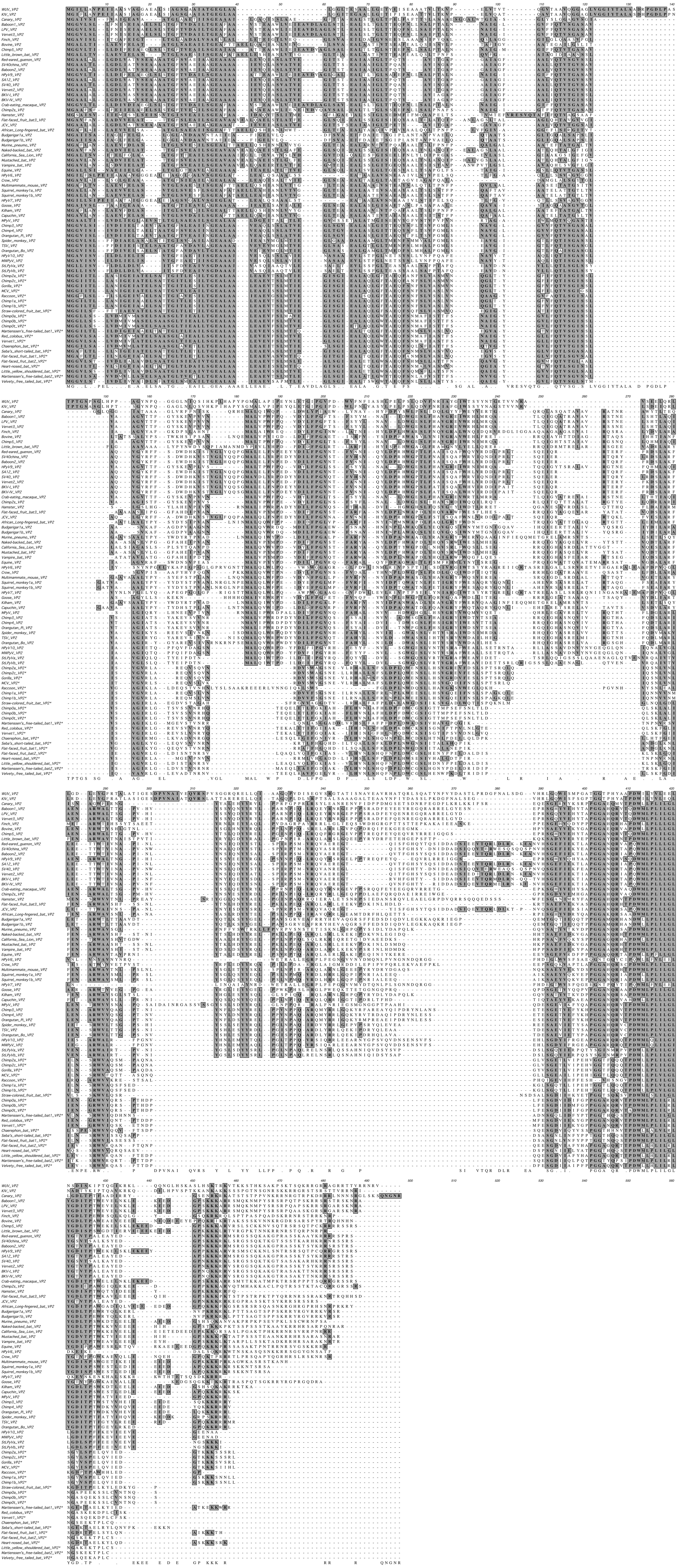

Supplement: Figure S1 — Alignment of polyomavirus VP2 proteins. A representative set of VP2 proteins from known polyomavirus species was aligned using MUSCLE with PAM200 matrix. The names of polyomavirus species that lack the conserved VP3 MALXXΦ N-terminus motif (position ∼180 of the alignment) are marked with an asterisk. (PDF) [file ppat.1003558.s001.pdf]
