# Supplementary figures and images for: The Merkel Cell Polyomavirus Minor Capsid Protein
Source: PLoS Pathog. 2013 Aug 22;9(8):e1003558. doi: 10.1371/journal.ppat.1003558 (PMC3749969; doi:10.1371/journal.ppat.1003558)

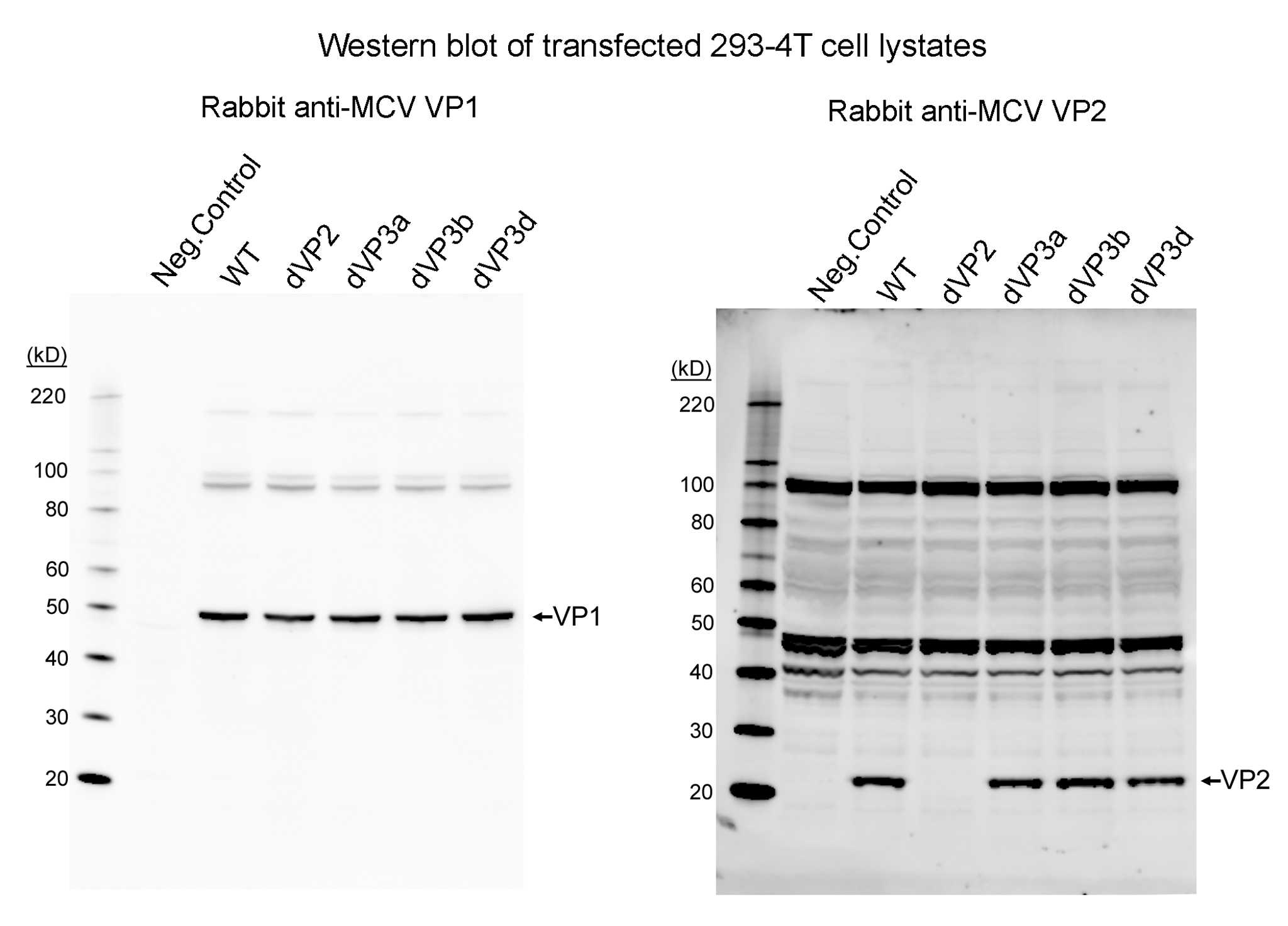

Supplement: Figure S2 — Viral protein expression from MCV genomic DNA in 293-4T cells. Whole cell lysates of 293-4T cells transfected six days prior with WT or mutant MCV genomic DNA were separated by SDS-PAGE and western blotted with rabbit polyclonal VP1 antiserum (left) or rabbit polyclonal VP2 antiserum (right). The VP2 blot reveals no VP3-like proteins in cell lysates. (TIF) [file ppat.1003558.s002.tif]

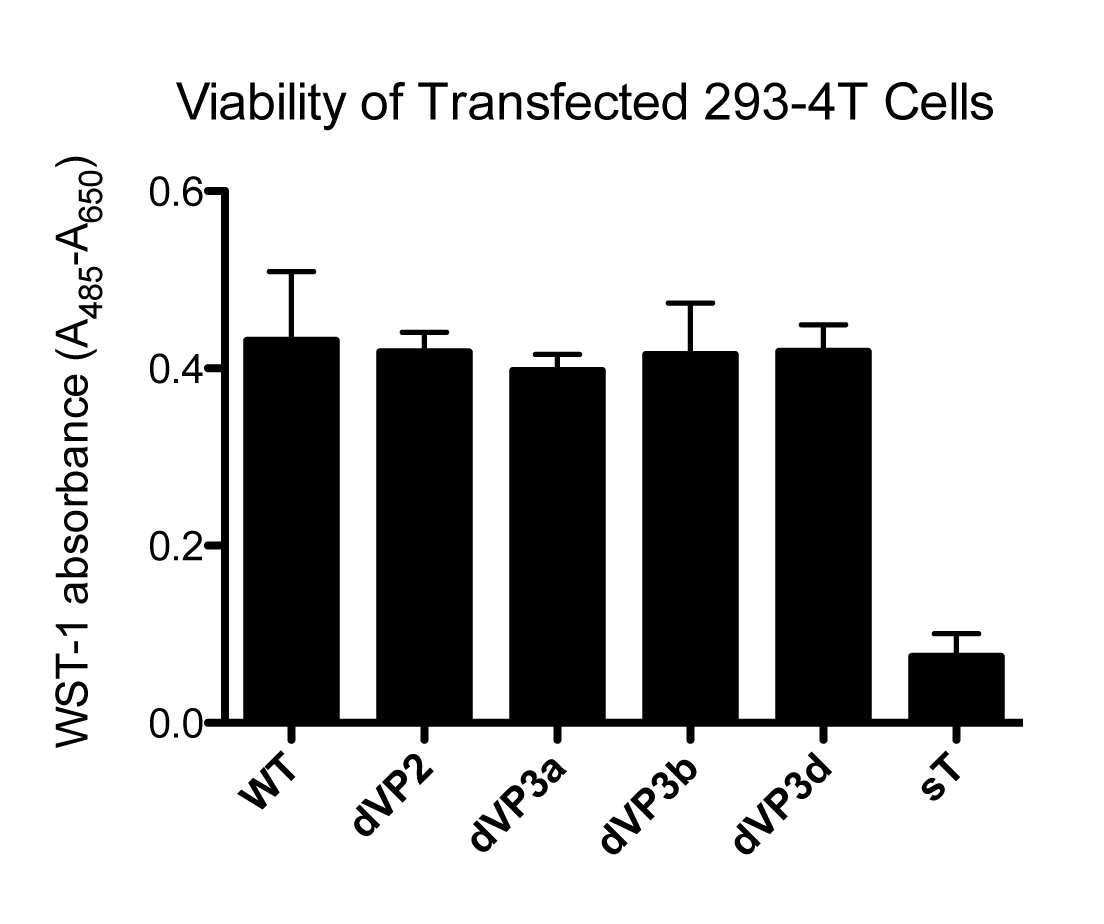

Supplement: Figure S3 — Viability of 293-4T cells transfected with MCV genomic DNA. WST-1 reagent was added to 293-4T cells transfected six days prior with WT or mutant MCV genomic DNA or a MCV small t antigen (sT) expression construct known to be cytotoxic. Absorbance was measured and averaged at multiple time points after WST-1 addition in triplicate wells. The average of three experiments is shown and error bars represent the standard error of the mean. (TIF) [file ppat.1003558.s003.tif]

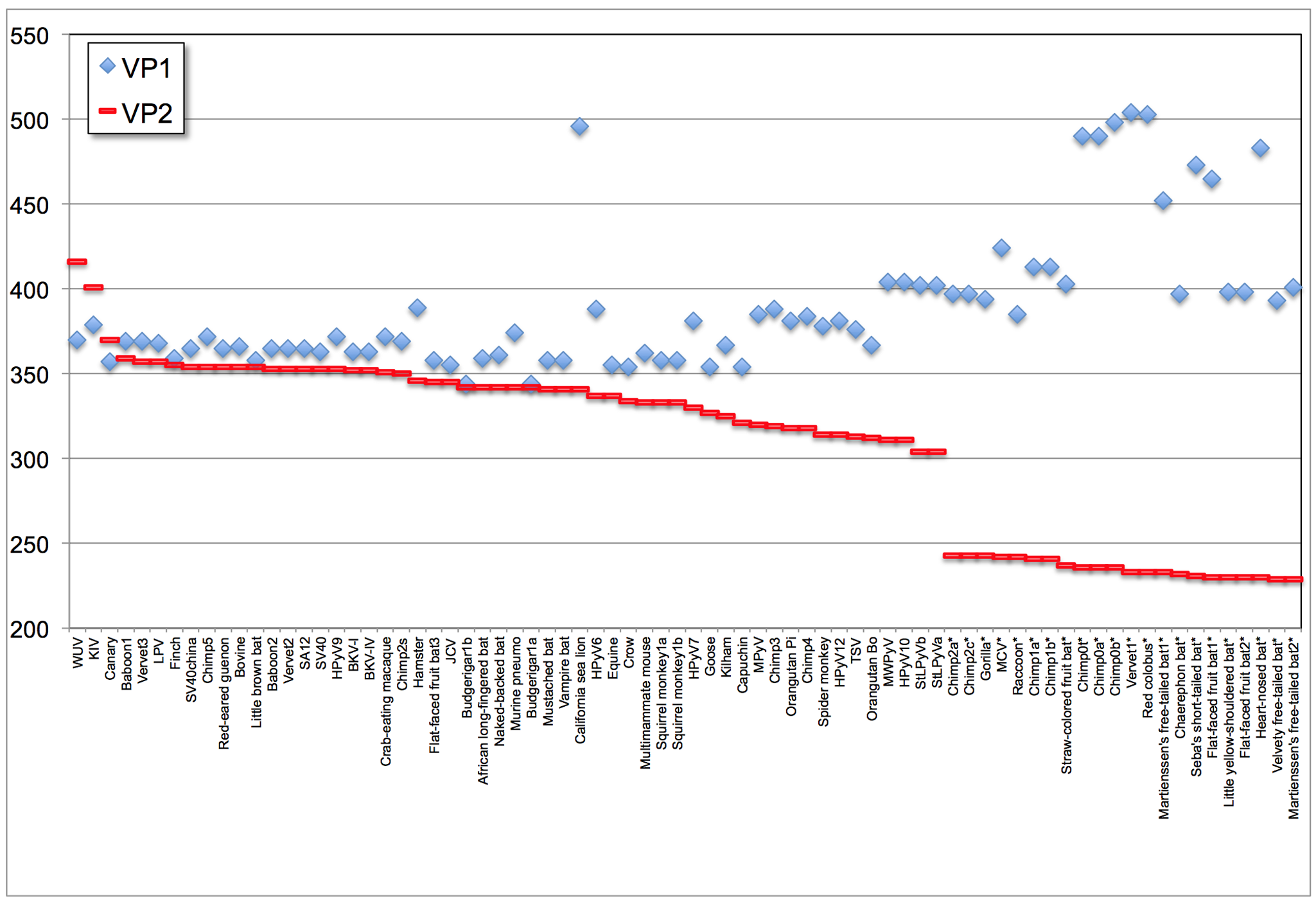

Supplement: Figure S4 — VP1 and VP2 ORF length. The y-axis shows the length (in number of codons) of the VP1 and VP2 ORFs for various polyomavirus species. The names of putatively VP3-less species are marked with asterisks. (TIF) [file ppat.1003558.s004.tif]

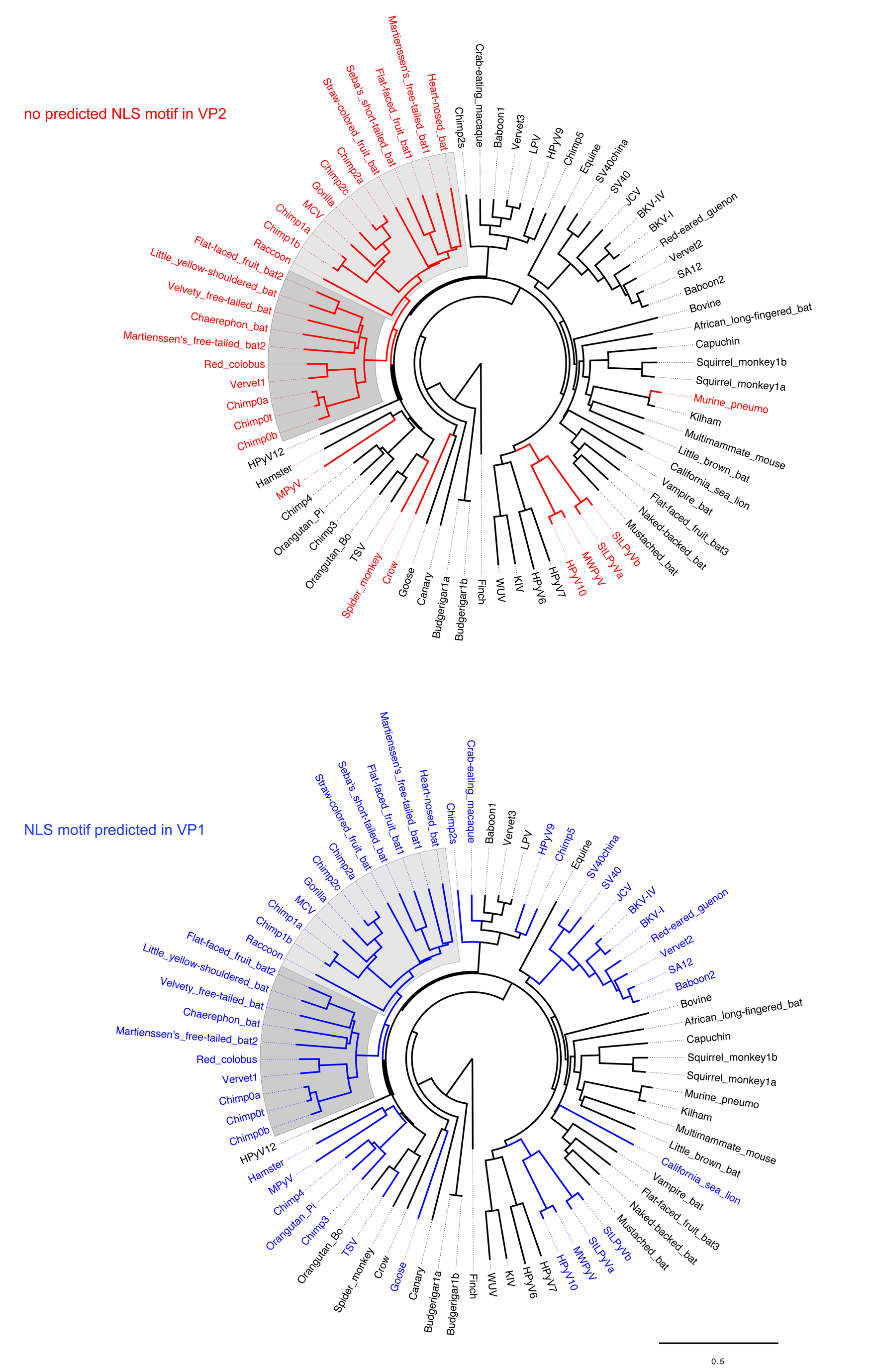

Supplement: Figure S5 — Polyomavirus phylogenetic trees displaying predicted nuclear localization sequences in VP2 or VP1. Polyomavirus species from the same neighbor-joining tree shown in Figure 12 are color-coded to indicate a predicted lack of NLS in VP2 (red) or presence of NLS in VP1 (blue). (TIF) [file ppat.1003558.s005.tif]
